# Emergent Chemical Behavior in Variable-Volume Protocells

**DOI:** 10.3390/life5010181

**Published:** 2015-01-13

**Authors:** Ben Shirt-Ediss, Ricard V. Solé, Kepa Ruiz-Mirazo

**Affiliations:** 1 ICREA-Complex Systems Lab Institut de Biologia Evolutiva CSIC-UPF 08003 Barcelona Spain; E-Mails: ben@shirt-ediss.me (B.S.-E.); ricard.sole@upf.edu (R.V.S.); 2 Logic and Philosophy of Science Department, University of The Basque Country, Avda. Tolosa 70, 20018 Donostia-San Sebastian, Gipuzkoa, Spain; 3 Santa Fe Institute, 1399 Hyde Park Road, Santa Fe, NM 87501, USA; 4 Biophysics Unit (CSIC, UPV/EHU), University of The Basque Country, Barrio Sarriena s/n, 48940 Leioa, Bizkaia, Spain

**Keywords:** semi-permeable compartments, osmosis, variable solvent volume, mass action kinetics (MAK), chemical reactor, continuous-flow stirred tank reactor (CSTR), bistability, systems chemistry, osmotic coupling

## Abstract

Artificial protocellular compartments and lipid vesicles have been used as model systems to understand the origins and requirements for early cells, as well as to design encapsulated reactors for biotechnology. One prominent feature of vesicles is the semi-permeable nature of their membranes, able to support passive diffusion of individual solute species into/out of the compartment, in addition to an osmotic water flow in the opposite direction to the net solute concentration gradient. Crucially, this water flow affects the internal aqueous volume of the vesicle in response to osmotic imbalances, in particular those created by ongoing reactions within the system. In this theoretical study, we pay attention to this often overlooked aspect and show, via the use of a simple semi-spatial vesicle reactor model, that a changing solvent volume introduces interesting non-linearities into an encapsulated chemistry. Focusing on bistability, we demonstrate how a changing volume compartment can degenerate existing bistable reactions, but also promote emergent bistability from very simple reactions, which are not bistable in bulk conditions. One particularly remarkable effect is that two or more chemically-independent reactions, with mutually exclusive reaction kinetics, are able to couple their dynamics through the variation of solvent volume inside the vesicle. Our results suggest that other chemical innovations should be expected when more realistic and active properties of protocellular compartments are taken into account.

## 1. Introduction

The rise of cellular life on the early Earth provided a unique opportunity for escaping from the vagaries of chemical interactions happening in a compartment-free context [1]. Once formed, lipid membranes or other types of prebiotic compartments (e.g., coacervates [2]) constitute the natural source of asymmetries that fundamentally characterize cells as non-equilibrium systems [3]. In that context, gradients and selective diffusion forces may start ruling the matter and energy flows between an internal distinct medium and the external environment. These flows are ultimately responsible for powering reactions that sustain cellular processes and structures. In our modern, evolved living world, cells exhibit a highly complex set of feedbacks and control mechanisms between biosynthetic reaction pathways and the dynamical organization of the membrane, including channels, receptors and complex supramolecular structures. On these lines, some authors [4] claim that metabolisms should no longer be conceived just as complex networks of cyclic, strongly-regulated and coupled reactions, but in addition, ought to be understood as intrinsically “vectorial” [5], *i.e.*, involving membrane processes and dynamics from their very core.

In this theoretical study, we take as a starting point a prebiotic scenario in which primitive protocells (lipid vesicles) would spontaneously form and behave as dynamic supramolecular structures that can host and get coupled to various chemical reactions within their inner aqueous medium. We shall focus on the non-linear cross-effects appearing when these chemically-active protocellular systems are treated as proper dynamic entities; in particular, when they are no longer considered to have a fixed volume. For that purpose, we develop a minimal semi-spatial mathematical model of a variable-volume vesicle reactor, in which reaction and compartment dynamics can affect each other, driven by the relatively rapid water exchanges between the inner and outer media. Although other processes (such as growth, competition, eventual reproduction and inheritance) are fundamental to understand the evolutionary role of protocells [6,7,8,9], our goal in this article is to begin exploring the potential for chemical innovation *in situ*, in vesicle compartments that do not necessarily divide and have any offspring [10]. More specifically, we are interested in how coupling both components of the protocellular organization (semi-permeable membrane and encapsulated chemistry) can allow, under some circumstances, to expand the space of possible steady states exhibited by the whole system, beyond what is associated with the pure reaction dynamics inside.

Before proceeding with the definition of our model, which is based on the original work of [11,12], we will briefly review other theoretical studies of compartmentalized chemical systems that have addressed, one way or the other, the question of inner volume variability. This survey will allow placement of our model within a wider context of ongoing research.

Variable solvent volume was obviously recognized early on as an important factor affecting, for example, catalytic activity [13], but it is only recently that a handful of studies paying attention to this aspect have started to appear. In 2004, a “dilution term” was introduced as a necessary addition in deterministic concentration ODEs to properly describe reactions in changing solvent volume [14], and more recently, this framework has been used to expose the unexpected character of enzymatic reactions happening inside changing volume cell organelles [15]. In the arena of stochastic modeling, and also in 2004, initial extensions to the Gillespie Stochastic Simulation Algorithm (SSA) were proposed to handle the simulation of reactions in a cell volume that periodically doubled and then divided [16].

With respect to protocells, the major shortcoming of the latter studies was that the system volume did not have an osmotic dependence on the concentrations of the reaction species involved. Rather, variations in the volume were conceived as a result of a deterministic process independent of the chemical reaction dynamics. The reason was that the reactions under study were thought to form small sub-networks of a much larger system whose complete dynamics were unspecified, but which was, nevertheless, known to change volume in a predictable way (e.g., through cell division).

More recently, Martín *et al.* [17] have used the dilution term to model primitive cells, where metabolic complexity is strongly reduced. In this protocell scenario, the complete description of the reaction system encapsulated within the membrane is known, and this permits the variation of volume to be formulated, via osmotic considerations, as explicitly depending on the total concentration of internal species. They revealed that well-studied chemical oscillators, when in variable volume “vesicle-like” conditions (even if the membrane as such is not modeled), showed altered limit cycles depending on how strongly the rate of change in volume was influenced by the rate of change in total solute concentrations. Degeneration to a single, stable steady state was generally observed for larger volume changes. On the stochastic front, an approach to extending the Gillespie SSA to vesicle systems whose volume changes, via water osmosis, as a function of internal reactant concentrations has been proposed [11,12,18]. Here, the assumption is made that vesicle volume updates instantaneously, with zero lag, after each event producing or consuming solute molecules inside the vesicle. This strategy leaves the core mathematics of the Gillespie algorithm unchanged by simply re-calculating the molecular event propensities from deterministic rate constants [19] after each event, taking into account the updated volume. Neglecting water osmosis, an alternative line of research has been to consider reactions inside variable surface area compartments instead and derive the compartment volume by assuming that the enclosing membrane is always spherical (for example, Villani *et al.* in this Special Issue [20], or models of Ganti’s chemoton [21,22,23]). In the stochastic domain, [24] have made an approximate reformulation of the Gillespie SSA to deal with reactions in a volume whose growth is dictated by a monotonically increasing spherical membrane.

While all of the models reviewed thus far acknowledge the relevance of changing volume to a cell or protocell system, it is quite remarkable that almost all neglect that solutes actually have to passively diffuse across a lipid bilayer surface in order to enter or leave the variable volume water pool. One recent semi-empirical study that does pay attention to how finite membrane diffusion rates could limit resources to a compartmentalized proto-metabolism (but within a fixed volume, fixed surface area vesicle) is [25]. Along the same lines, our own previous work has performed a theoretical analysis of a very simple “bioreactor” consisting of a unimolecular reaction happening inside a vesicle, where the vesicle has again fixed surface area, but now variable volume and mechano-sensitive channels in the membrane [26]. The vesicle reactor model of the current study is a significant extension of this latter model, further incorporating a first approach to variable vesicle surface area and considering more complex reaction chemistries inside the vesicle reactor.

All studies briefly reviewed above, including the present study, fall within the category of semi-spatial, “well-stirred” compartment models. Under this simplifying approach, diffusion of solutes only occurs across membrane interfaces and is otherwise considered instantaneous throughout solution phases. However, it is important to mention that fully spatially explicit models of compartmentalized chemistries inside vesicles have also been developed. In particular, Macía and Solé [27] presented a two-dimensional vesicle model in which a membrane-bound “Turing” reaction-diffusion system was able to exert non-uniform osmotic pressures along the surface of the (spatially resolved) membrane, leading to spontaneous and indefinitely repeatable vesicle division. In another recent work [28], a two-dimensional hypothetical vesicle was instead modeled as a one-dimensional periodic array of micro cells, spatially representing the portion of solution contacting the vesicle membrane on the interior side. Stochastic simulation of this model with autocatalytic reactions lead to oscillations between microcells, again providing non-uniform osmotic pressures along the surface of the membrane.

With respect to these advanced studies, our current contribution can be regarded as a minimal model to demonstrate, in the simplest possible way, the consequences that a changing solvent volume may have on a set of encapsulated reactions. The article is structured as follows. First, in the Reactor Models section, two classical bulk reaction scenarios are introduced, which will be later used for comparative purposes. Then, the vesicle reactor model is described, followed by a qualitative graphical method used to find its fixed points. The Results section examines three case studies, where it is shown that our treatment of the problem to encompass variable-volume conditions does have relevant effects on the internal chemical dynamics. More concretely, multiple states are found to emerge from the coupling of reactions and variable volume provided by a surrounding semi-permeable membrane. Here, the new idea of “osmotic coupling” of reactions is explained in detail. The Discussion section summarizes the significance of our results, recapitulates limitations and suggests future research directions to explore. Finally, the Methods section outlines the strategy used to tackle the large parameter space of the vesicle reactor model, a strategy indispensable in producing the main results. The Supplementary Material (on line) contains essential supporting derivations and data.

## 2. Reactor Models

**Figure 1:**
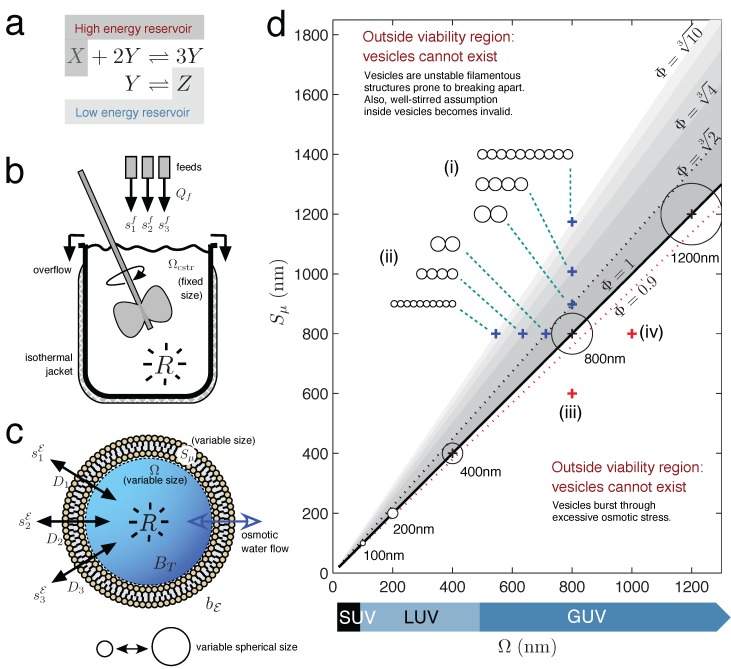
Chemical Reactor Models. (**a**) A chemical reaction held far-from-equilibrium by use of two constant concentration reservoirs (reservoir conditions). (**b**) Continuous-flow stirred tank reactor (CSTR) model with non-limiting outflow, constant solvent volume and constant reaction temperature. (**c**) Unilamellar lipid vesicle, with variable internal solvent volume determined by osmotic water flow equalizing the total solute concentration difference across the semi-permeable membrane. (**d**) Lipid vesicle morphology space, with vesicle viability space drawn as the sub-region of all possible morphologies (grayscale region). Axes express vesicle volume Ω and surface area *S_µ_* as the nm diameter of a sphere having volume Ω or surface area *S_µ_*, respectively. Spherical vesicles (Φ = 1) are located when Ω (in nm)= *S_µ_* (in nm). Circles represent vesicle cross-sections, drawn to scale (8.5% of the axes’ scale). Blue crosses indicate how the morphology of a spherical d = 800 nm giant unilamellar vesicle (GUV) changes, when (i) gaining surface or (ii) losing volume. Both conditions bring the vesicle toward more filamentous or prolate states. Prolate states are depicted as strings of smaller spherical vesicles, only to give an idea of the volume and surface ratio at the {Ω*, S_µ_*} point; the meaning is not that the vesicle has necessarily divided into identical daughters at this stage. Red crosses indicate that the spherical d = 800 nm vesicle will burst by (iii) losing too much surface or, conversely, (iv) gaining too much volume.

The traditional and mathematically most simple way to formalize a reaction system held far from equilibrium (FFE) is to assume well-stirred conditions and the existence of a permanent concentration gradient in the form of two (or more) inexhaustible reservoir species (Figure 1a). Under these reservoir conditions, the dynamic behavior of reaction intermediate species is of interest, species which dissipate energy between the high and low energy reservoirs (resource and waste species).  Such a system has a dimension equal to the number of intermediate species only, and each concentration time derivative contains just mass action kinetics (MAK) terms.

However, actually constructing a chemical reactor that implements the reservoir conditions outlined above seems problematic. In order to maintain the chemical system in an FFE state indefinitely, the constant resource-waste concentration gradient would have to exist in an infinite reservoir, and an infinite reservoir would not allow the concentrations of the intermediate species to be measured. In terms of an actual chemical apparatus that is routinely used to carry out sustained FFE chemical reactions, the continuous-flow stirred tank reactor (CSTR) stands as one of the simplest devices [29]. Figure 1b shows the schematic of one of several CSTR designs available, a design that features a constant reaction volume due to a non-limited overflow of reactor effluent. A mechanical stirrer keeps the solution well-mixed, despite its potentially large volume. A system of reactions taking place in this type of CSTR, depending on the specific reaction kinetics involved, has a dimension up to the total number of species *N* [30]. If the reactor is isothermal, the dynamic concentration behavior of the *i*-th solute species is described by:

(1) $$\frac{ds_{i}}{dt}=r_{i}(\vec{s})+\;\frac{1}{\theta }(s_{i}^{f}-s_{i})$$

where $\vec{s}$ = {*s*_1_*,* *· · ·* *, s_N_*} is the vector of all species concentrations inside the reactor, function $r_{i}(\vec{s})$ contains all reaction MAK terms producing or consuming the *i*-th species, $s_{i}^{f}$ is the constant concentration of species *i* in the reactor feed pipe and *θ* is the mean residence time of the CSTR. The mean residence time is defined as the ratio of reactor solvent volume (constant) divided by the flow rate of solvent into the reactor *θ* = Ω_CSTR_*/Q_f_* and represents that solutes are “washed out” faster from a reactor having a higher inflow rate or a smaller volume.

The two previous scenarios may be described as “bulk conditions” for chemical reactions, whereby a relatively large homogeneous solution exists for chemical transformations to take place. As stated, in this work, we begin to investigate the behavioral possibilities that minimal chemical reaction systems can have inside variable-volume unilamellar lipid vesicle reactors. Figure 1c shows the full schematic of the vesicle reactor model, which is drawn more simply in the following figures as a clear sphere with a blue arrow crossing the membrane, depicting the important feature of osmotic water flow.

A lipid vesicle departs from bulk conditions as it partitions a solution into at least two heterogeneous phases (inside and outside), introducing structure into a chemical reaction medium [31]. As mentioned, the aqueous phase inside a lipid vesicle might provide a unique space for chemical reactions because of its variable solvent volume, following as a consequence of osmotic water flow through the surrounding semi-permeable lipid bilayer membrane. This osmotic flow quickly equilibrates the total solute concentration gradient existing between the vesicle interior and exterior, with water molecules moving across the membrane in the opposite direction to the net solute gradient. The bilayer also implies finite passive diffusion rates for permeating solutes.

To elaborate the details, the vesicle reactor is a system with a dimension equal to the total number of solute species *N* [32]. We assume that the aqueous volume of the vesicle changes instantaneously, such that total internal and external solute concentrations remain always equal (isotonic condition). Therefore, this volume is given directly by the following function:

(2) $$Ω=\frac{B_{T}}{C_{\varepsilon }-\sum_{j=1}^{N}s_{j}}$$

where Ω is the scaled volume Ω = *N*_*A*_*𝒱* (Avogadro’s constant multiplied by the liter volume of the vesicle), *B*_*T*_ *>* 0 is the number of non-reacting impermeable buffer molecules trapped inside the vesicle [33], *C*_*ε*_ is the total external concentration, that is the sum of all the external solute concentrations $C_{\varepsilon }=b_{\varepsilon }+\sum_{j=1}^{N}s_{j}^{\varepsilon }$, and $\sum_{j=1}^{N}s_{j}$ is the total internal concentration of chemically-reacting solute species inside the vesicle. The assumption of instantaneous vesicle volume change is based on the observation that water permeates a fatty acid membrane on a time scale that is orders of magnitude faster than the passive diffusion of solutes [34] and simplifies the model, because it does not require treating Ω as an extra state variable.

For the surface area kinetics of our vesicle reactor model, as a first approximation, we assume that the membrane surface area immediately follows changes in vesicle volume, maintaining a spherical shape, *i.e.*:

(3) $$S_{µ}=K{Ω}^{\frac{2}{3}}$$

where constant $K={\left(\sqrt{36\pi }/N_{A}\right)}^{\frac{2}{3}}$. Under these conditions, an increase in internal vesicle volume is considered to be instantly accompanied by lipids associating with the bilayer from the surrounding solution, flip-flopping and enlarging the surface of the vesicle, with the reverse process happening for a decrease in internal volume. This simplification avoids having to explicitly specify lipid exchange kinetics for the membrane, which can be complex [9] and would further introduce extra state variables into the model.

Initially, one may consider the vesicle model under high buffer conditions, which is the limit case of the external buffer concentration *b*_*ε*_ being very high with respect to the possible range of external and internal solute concentrations (*b*_*ε*_ $\gg s_{j}^{\varepsilon }$ and $b_{\varepsilon }\gg s_{j}$). Under such conditions, there is little net water movement across the membrane, and the vesicle volume is approximately constant at ${Ω}^{\emptyset }\approx B_{T}/b_{\varepsilon }$. Assuming well-stirred kinetics within the vesicle water pool [35], the dynamic behavior of the concentration of the *i*-th solute species inside the vesicle would be described by:

(4) $$\frac{ds_{i}}{dt}=r_{i}(\vec{s})+\frac{1}{{Ω}^{\emptyset }}S_{\mu }^{\emptyset }D_{i}(s_{i}^{\varepsilon }-s_{i})\;$$

where $S_{\mu }^{\emptyset }$ is the constant membrane surface area corresponding to the approximately constant volume ${Ω}^{\emptyset }$ and *D_i_* is the diffusion constant for species *s*_*i*_ calculated as:

(5) $$D_{i}=\frac{D_{i}^{\times }D_{\text{ribose}}}{\lambda }$$

where diffusion constants in this work are specified more meaningfully as multipliers ($D_{i}^{\times }$) to the diffusion constant that ribose has through an oleic acid membrane *D*_ribose_ = 2*.*65 *×* 10^8^ dm^2^ s*^−^*^1^ mol*^−^*^1^ [11], and the vesicle bilayer is considered to have constant thickness *λ* = 4 *×* 10*^−^*^8^ dm. Fick’s law provides the basis for the membrane diffusion term.

It is useful to review the high buffer case of the vesicle reactor, since the reservoir and CSTR reaction scenarios described previously can both be interpreted as theoretical limit cases of it. In other words, the vesicle reactor model is the general case of the reaction scenarios shown on Figure 1. Reservoir conditions for an internal reaction in the vesicle model in a high buffer regime can be ensured if the diffusion constants for resource and waste species are sufficiently high, so that any variation in internal concentration is quickly equilibrated back to the respective constant external concentration, and conversely, the diffusion constants for the intermediate species are set to zero. Likewise, CSTR conditions are reproduced when all species have an identical diffusion constant *D*, and external vesicle solute concentrations are set to the CSTR feed concentrations $s_{i}^{\varepsilon }=s_{i}^{f}$ [36]. Then, the CSTR mean residence time is related to the vesicle reactor parameters by:

(6) $$\theta =\frac{B_{T}}{S_{µ}^{\emptyset }Db_{\varepsilon }}$$

Nevertheless, in this work, we precisely aim to relax the high buffer assumption and study chemical behavior in the vesicle reactor under low buffer conditions, when the concentrations of diffusing solutes are in the same order of magnitude as the concentration of external buffer. This moves the model well away from reservoir and CSTR conditions, providing a very different context for internal reactions. In low buffer conditions, a significant net movement of water across the vesicle membrane must be accounted for, and hence, the aqueous volume inside the vesicle must be considered variable. This variable volume introduces several non-linear terms into the solute concentration derivatives, as can be seen below, providing a rich substrate for emergent chemical behavior in the vesicle reactor. The concentration derivatives specifically change to:

(7) $$\frac{ds_{i}}{dt}=r_{i}(\vec{s})+S_{µ}\frac{D_{i}}{B_{T}}\left(C_{\varepsilon }-\sum_{j=1}^{N}s_{j}\right)(s_{i}^{\varepsilon }-s_{i})-\frac{s_{i}dΩ}{Ωdt}$$

where *S_µ_* is now variable and given by Equation (3). The above expression is formed by substituting Equation (2) into Equation (4) and then adding the dilution term [14] to properly account for changing volume in a concentration ODE. Considering that vesicle volume is a function of internal solute concentrations Equation (2), the dilution term is given by:

(8) $$-\frac{s_{i}}{Ω}\frac{dΩ}{dt}=-\frac{s_{i}}{C_{\varepsilon }}\sum_{j=1}^{N}\left(r_{j}(\vec{s})+\frac{1}{Ω}S_{µ}D_{j}(s_{j}^{\varepsilon }-s_{j})\right)$$

which is derived and explained in detail in the Supplementary Material. On the subject of emergent chemical behavior, one important observation following from the fact that each derivative is now a function of all solute concentrations $\vec{s^{*}}$ is that chemically-independent reaction sets sharing the vesicle volume will become “osmotically” coupled. This aspect is explored in detail later.

To narrow its scope, this work will just analyze the fixed points of the ODE set Equation (7) and only for very simple reaction networks encapsulated inside the vesicle reactor. A fixed point is a special set of internal solute concentrations $\vec{s^{*}}$ at which all solutes have no further change in their concentrations, *i.e.*, *ds_j _/dt* = 0*, j* = 1*, . . . , N*. In this state, the vesicle volume and surface are stationary.

In particular, in these initial stages, we will focus on the emergence of bistability in the vesicle reactor model, a dynamical feature deducible directly from the number and stability of the fixed points present (*i.e.*, two asymptotically stable points separated by an unstable saddle point). We also expect that more complicated dynamical regimes could also be present in the model, like multi-stability or global phase space features, such as limit cycles, giving rise to sustained oscillations. However, the investigation of these regimes will be deferred to later work: for the time being, the “emergent chemical behavior” referred to in the title will be restricted to bistability.

The following two subsections describe a graphical method used for seeing, in a qualitative way, what fixed point solutions to Equation Set (7) can exist. These subsections introduce the concepts of vesicle morphology space, bifurcation curve and vesicle viability space, which are necessary to properly understand the results presented in Section 3, and the Discussion.

### 2.1. Solution of Vesicle Reactor Model: Graphical Method

In trying to solve fixed points of the variable volume vesicle reactor model ODE Equations (7), it can be observed that the dilution terms Equation (8) can be usefully disregarded, since the vesicle volume is not changing at steady state (*d*Ω*/dt* = 0). However, whilst this provides a significant simplification, the *S_µ_* term in Equation (3) still makes the remaining equations difficult to solve for zeros, even numerically.

We proceed by relaxing the need for exact fixed point solutions to Equation (7). Instead, we pursue a graphical approach that pictorially shows how many fixed point solutions will exist to the equation set (and the approximate values of those fixed points), given a certain parameter regime. This graphical approach uses the following algorithm:

**Step 1: **The fixed points for a variable volume vesicle reactor with constant surface $S_{\mu }^{\emptyset }$ are solved. With constant surface, the concentration derivatives at fixed points simplify to a set of multivariate polynomials in the species concentrations:

(9) $$\frac{ds_{i}}{dt}=r_{i}(\vec{s})+S_{\mu }^{\emptyset }\frac{D_{i}}{B_{T}}\left(C_{\varepsilon }-\sum_{j=1}^{N}s_{j}\right)(s_{i}^{\varepsilon }-s_{i})=0$$

This equation set is solved numerically by a polynomial homotopy continuation procedure ([37], see the Methods section). There may be zero or many fixed points present. Indeed, one interesting aspect is that its not obvious from inspection of Equation (9) what the limit number of fixed points will be, even for a simple reaction system inside the vesicle reactor.

**Step 2: **The solute concentrations at each fixed point are converted to the corresponding fixed point vesicle volume Ω^∗^, using Equation (2). Then, all of the fixed point volumes are plotted on a two-dimensional graph, which we call the vesicle morphology space, where the x-axis represents vesicle volume and the y-axis represents vesicle surface (Figure 1d). The fixed point volumes are plotted along horizontal line *y* = $S_{\mu }^{\emptyset }$in this space.

**Step 3: **Vesicle surface $S_{\mu }^{\emptyset }$ is incremented, and the process repeated from Step 1.

The above algorithm effectively assesses a series of fixed surface vesicles to build up “branches” of fixed point solutions, which run throughout the vesicle morphology space. We will call this the “bifurcation curve” throughout the text, since it tracks the existence and locations of fixed point locations of Equation Set (9), under variation of control parameter *S_µ_*.

Crucially, the branches of the bifurcation curve define different (not necessarily spherical) vesicle shapes and sizes {Ω*, S_µ_*} that allow the encapsulated reaction network to reach steady state, for a given model parameter set. The fixed point solutions to the full reactor model Equation (7) are precisely where the branches of the bifurcation curve intersect the line, giving spherical vesicle morphologies (Φ = 1 line; see the next subsection). Local stabilities of fixed points can also be calculated at Step 1. However, at best, these are “quasi-stabilities” or predictors of stability in the full model, because the dilution term and the fact that surface area is actually variable are both not taken into account.

Now that an approximate method to find the fixed points of the full vesicle model Equation (7) has been established, the second problem involves finding working parameter regimes, in the high-dimensional parameter space of the model, which will give three crossings of the spherical morphologies line (*i.e.*, potentially corresponding to bistability in the full model). The Methods section at the end of the article defines our approach to this non-trivial “needle and haystack” issue.

### 2.2. Vesicle Viability Space within Vesicle Morphology Space

Figure 1d draws the vesicle morphology space for unilamellar vesicles ranging from small unilamellar vesicles (SUV) to giant unilamellar vesicles (GUV) and additionally draws the vesicle viability space sub-region (colored grey). The vesicle viability sub-region arises because lipid vesicles are soft supra-molecular structures held together by entirely non-covalent forces and, as such, can only provide an internal aqueous domain for a restricted set of volume and surface area combinations. The vesicle viability region is calculated from simple geometric considerations, by defining a dimensionless ratio called reduced surface:

(10) $$\Phi =\frac{S_{µ}}{\sqrt[{3}]{36\pi {\left(Ω/N_{A}\right)}^{2}}}$$

When Φ = 1, a unilamellar vesicle is spherical (the surface area wraps the volume as a sphere), whereas Φ *>* 1 represents a deflated vesicle (surplus surface area) and Φ < 1 a vesicle in osmotic tension. Vesicles cannot venture too far into the osmotic tension region before bursting (Φ ≈ 0.9; see [11]), which provides a hard lower limit on valid vesicle morphologies. At the other end of the Φ scale, vesicles cannot become excessively filamentous structures before becoming prone to division into smaller structures. The upper Φ limit for vesicle morphologies is less well defined that the lower Φ limit and is better thought of as an increasing probability to divide rather than an absolute cut-off (hence the fading grey scale bars in Figure 1d. Lines for $\Phi =\sqrt[{3}]{2},\sqrt[{3}]{4},\sqrt[{3}]{10}$ indicate vesicles becoming more filamentous, not any absolute divide limit).

Even if vesicle viability space is not directly relevant to the vesicle reactor model Equation (7) in this present study (since the vesicle maintains a valid spherical Φ = 1 state at all times), it is nevertheless a concept relevant in the wider implications of this work (see the Discussion).

## 3. Results

In this section, we perform three case studies that assess the behavior of basic chemical reaction sets inside the variable volume vesicle reactor model. The first case study takes two reaction mechanisms, which are bistable in bulk conditions, and illustrates how encapsulation inside a vesicle water pool may trigger the disappearance of this property of bistability. The second and third case studies support the opposite scenario, whereby very simple reactions, which are not bistable in bulk, show emergent bistability when encapsulated inside the variable volume reaction environment of the vesicle model. Case Studies 2 and 3 convey the main message of this paper.

### 3.1. Case Study 1: Compartment as a Bottleneck to the Internal Reaction System

A lipid membrane undoubtedly provides a barrier to the free movement of solutes in and out of the vesicle interior. A common conception is that this barrier has a suffocating influence on an internal reaction by limiting the supply of nutrients and preventing the escape of waste products, such that the interesting chemical behavior originally present in the reaction under bulk conditions may degenerate [25]. This case study demonstrates that degeneration is, indeed, a real possibility. Two previously studied minimal reaction systems, which are bistable in reservoir conditions, are encapsulated in the vesicle reactor model and assessed for stability.

Firstly, the Schlögl model [38,39] was encapsulated. In reservoir conditions and permitting reversible tri-molecular reactions, this is the simplest chemical model to show bistability. It is one-dimensional, since there are just two reactions involving one intermediate species *Y* :

(11) $$X+2Y\overset{k1}{\underset{k_{1}^{r}}{⇌}}3Y\;\;\;\;\;\;\;Y\overset{k_{2}}{\underset{k_{2}^{r}}{⇌}}\;Z$$

One of the many possible parameter regimes leading to bistability in reservoir conditions is given by $\left\{x,z,k_{1},k_{1}^{r},k_{2},k_{2}^{r}\right\}$ = {6*.*47*×*10^*−*4^*,* 6*.*32*×*10^*−*4^*,*5*.*25*×*10^4^*,*2*.*85*×*10^*−*3^*,*9*.*15*×*10^*−*2^*,*7*.*15*×*10^*−*3^}, where concentrations are in molar, first order reaction rate constants in s^*−*1^ and third order in M^*−*2^s^*−*1^. The following molar concentrations represent the low stable state, the unstable state and the high stable state of species *Y* respectively: $\left\{y_{1}^{*},y_{u}^{*},y_{2}^{*}\right\}$ = {5*.*03*×*10*^−^*^5^*,* 4*.*01*×*10*^−^*^3^*,*7*.*86*×*10*^−^*^3^}.

The reaction system was incorporated inside the constant surface vesicle reactor model by setting the external solute concentrations to the original reservoir concentrations (*x*_*ε*_ = *x* and *z*_*ε*_ = *z*) and using the same reaction rate parameters. The remaining parameters were assigned at random in 5000 different combinations, in ranges given in the Methods section. The vesicle surface area $S_{\mu }^{\emptyset }$ was fixed at that of a 400-nm diameter sphere.

The number of fixed points present in the constant surface reactor was taken as a heuristic for the number that could exist in the full variable volume (and variable surface) vesicle reactor. If the reaction gave three fixed points, then three individual bifurcation branches passed through line $S_{\mu }^{\emptyset }$ in the vesicle morphology space at some stage, and these branches had the potential of being manipulated to cross through the Φ = 1 line and vesicle viability region.

Of the 5000 parameter set tested under constant surface area, 82% (4098) gave a single fixed point, 15.7% (785) gave two fixed points and 2.3% (117) no fixed points. No parameter regimes giving three fixed points (a bifurcation curve with three branches) were found in this sparse Monte-Carlo parameter sampling, and therefore, this dynamical feature of the reaction appears to have degenerated.

Secondly, the bistable Wilhelm model [40] of four irreversible reactions was encapsulated in the vesicle model

(12) $$\begin{array}{c} X+Z\overset{k_{1}}{\to }2Y\\ 2Y\overset{k_{2}}{\to }Y+Z\\ Y+Z\overset{k_{3}}{\to }Z+W\\ Y\overset{k_{4}}{\to }W \end{array}$$

Under reservoir conditions, one possible parameter regime giving bistability is {*x, w, k*_1_*,* *k*_2_*,* *k*_3_*,* *k*_4_}= {1*.*17*×*10^*−*3^*,* any*,* 5*.*86*×*10^2^*,* 9*.*26*×*10^2^*,* 5*.*75*×*10^2^*,* 9*.*98*×*10^*−*2^} with units the same as before and second order reaction rates in M^*−*1^ s^*−*1^. The following three solution pairs (in molar concentrations) for fixed points of the intermediate species are obtained: $\left\{y_{1}^{*},z_{1}^{*}\right\}$ = {0*,* 0}, $\left\{y_{u}^{*},z_{u}^{*}\right\}$ = {1*.*20*×*10^*−*4^*,* 1*.*94*×*10^*−*5^}, $\left\{y_{2}^{*},z_{2}^{*}\right\}$ = {1*.*07*×*10*^−^*^3^*,* 1*.*55*×*10*^−^*^3^}.

When the reaction system was incorporated inside the constant surface vesicle reactor model, of the 5000 random parameter sets sampled, 74.3% (3716) gave one fixed point, 20.9% (1045) gave two fixed points, 3.8% (192) no fixed points and <1% (47) gave three fixed points. Therefore, in this case, a very small proportion of the parameter sets showed potential to exhibit bistability in the full vesicle model, but the majority did not.

In summary, these results seem to demonstrate that only a very small proportion of parameter space is able to create bistability in the full vesicle model and that encapsulated reaction schemes are likely to degenerate to a single fixed point. However, this hypothesis cannot be easily confirmed, since a systematic exploration of parameter space is virtually impossible due to the curse of dimensionality. Thus, it could be argued that the random Monte-Carlo sampling of such a high dimensional space was far too sparse to be truly representative of the fixed point motifs possible for each of the switch reactions above.

**Figure 2:**
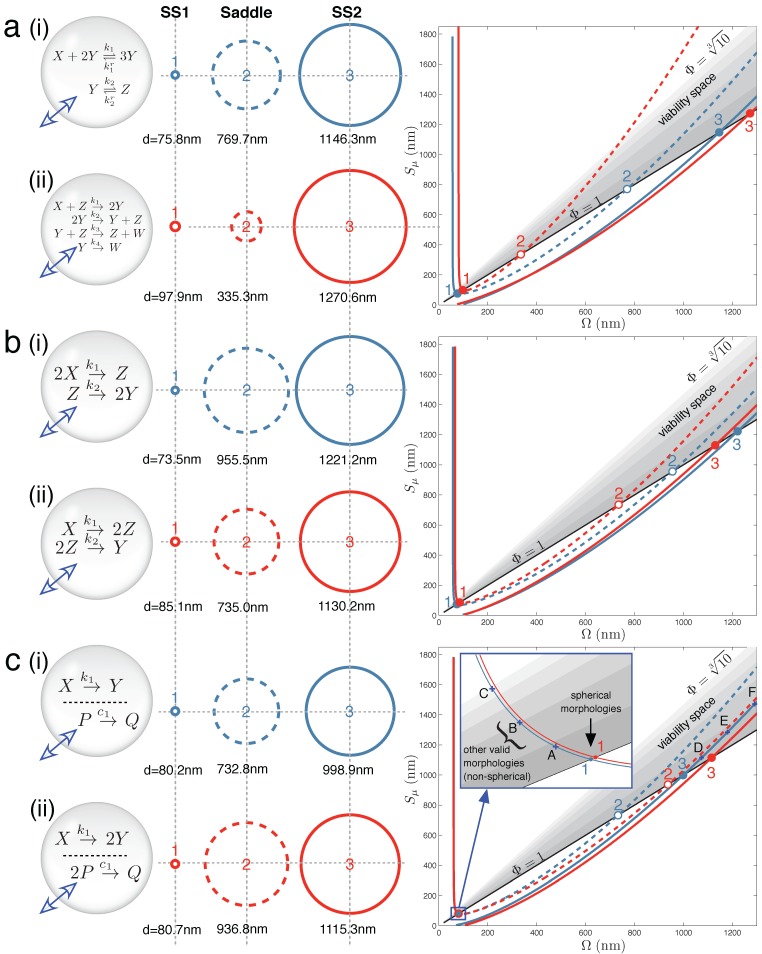
Main results: vesicle bistability in Case Studies 1–3. The reaction sets of (**a**) Case Study 1, (**b**) Case Study 2 and (**c**) Case Study 3 can yield bistability in the vesicle model under suitable parameter conditions (given in Supplementary Material). Circles (**middle**) depict spherical vesicle shapes (drawn to scale) at which steady states can occur, corresponding to where stable branches (solid lines) and unstable branches (dashed lines) on the bifurcation diagrams (**right**) cross the the Φ = 1 line. Labels A–F on the bifurcation diagram (c) refer to non-spherical morphologies drawn in Figure S2 of the Supplementary Material.

**Figure 3:**
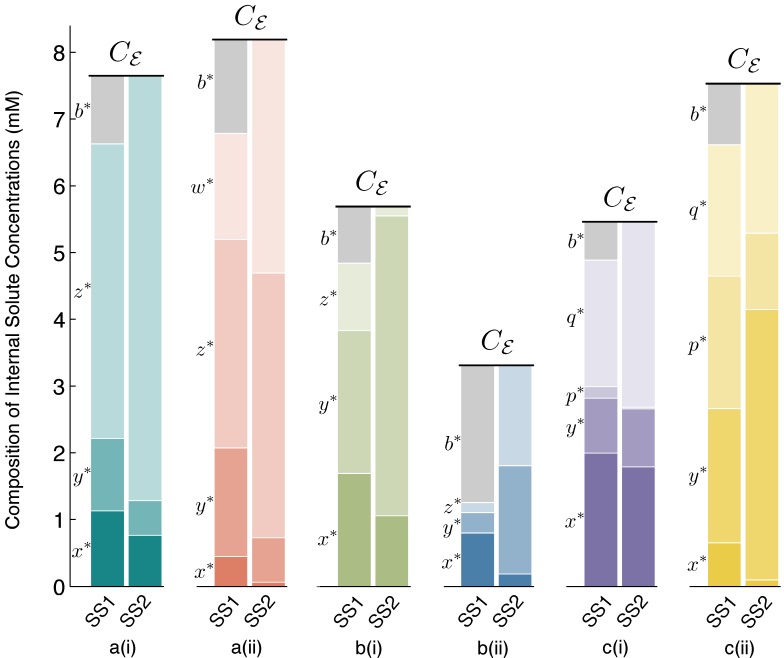
Steady State 1 (SS1) and SS2 internal solute concentrations. Solute concentrations inside the vesicle reactor, at spherical steady states SS1 and SS2, for each reaction scheme reported in Figure 2. Comparing SS1 and SS2 for each scheme, it can be observed that there are quantifiable differences in solute concentrations between steady states, but these differences are fairly small. Owing to the osmotic water balance, the total concentration of solutes inside the vesicle (height of the stacked bars) is always constrained to be equal to the total external solute concentration of the environment, *C_ε_*. Thus, in the vesicle reactor model, the main feature distinguishing steady states is vesicle size (see Section 4.2 for details). Symbol *b^∗^*denotes the steady-state concentration of the *B_T_*buffer molecules trapped inside the vesicle. At SS2, *b^∗^→* 0, due to large vesicle sizes, and the diffusing solutes constitute the majority of total internal concentration. The Supplementary Material supplies data supporting the figure.

In contrast, the following two case studies provide counterexamples that support the opposite scenario, in which interesting emergent chemistry may develop as a consequence of the volume-changing aqueous interior of a vesicle. Figure 2 displays the main results for Case Studies 1–3. It demonstrates that under the correct parameter regimes, each of the two reaction schemes discussed in each case study can show bistability within the full vesicle model. Vesicle morphology space and bifurcation curves are drawn for each reaction scheme. Even the Schlögl and Wilhelm reaction systems of the present case study can exhibit bistability for some parameters (Figure 2a(i) and 2a(ii), respectively). Figure 3 accompanies Figure 2, to make clear the concentration values of all internal solutes at stable steady states SS1 and SS2 for each of the six schemes.

### 3.2. Case Study 2: Compartment as Enabling New Steady States for Single Reaction Sets

In this case study, two very simple reaction sequences are demonstrated to be capable of bistability under specific parameter regimes of the vesicle reactor model (the Methods section details how these parameter schemes were obtained). Conversely, these reaction sequences can be easily proven to lack bistability in reservoir conditions and in CSTR conditions, under any parameter regime (see Supplementary Material). Irreversible reaction sequences were chosen intentionally for their fairly straightforward steady-state calculations in CSTR; reactions involving feedbacks are typically very difficult or impossible to solve analytically in CSTR flow conditions.

Figure 2b(i) shows one instance of the following second order-first order reaction sequence:

(13) $$2X\overset{k_{1}}{\to }Z\overset{k_{2}}{\to }2Y$$

in which bistability was found in the context of the vesicle model. Likewise, Figure 2b(ii) shows one instance of the following first order-second order reaction sequence:

(14) $$X\overset{k_{1}}{\to }2Z\overset{k_{2}}{\to }Y$$

that also demonstrates bistability. In each case, three crossings of vesicle viability space are obtained by the bifurcation curve. In the full vesicle model, this translates into a stable spherical state at a small SUV vesicle size (Ω and *S_µ_* *≈* 80 nm diameter) and another stable state at a much larger GUV vesicle size (Ω and *S_µ_* *≈* 1200 nm diameter). To re-iterate, the stable steady state means that the vesicle sphere is providing the correct diffusion surface and inner volume for all of the solute concentrations in the reaction network to be stationary, and at the same time, the total concentration of solutes and buffer inside the vesicle is equal to the total external concentration; and so, there is no net movement of water across the bilayer membrane. The steady-state sizes are separated by an unstable saddle point at an intermediate vesicle size.

### 3.3. Case Study 3: Compartment as Osmotically Coupling Two Chemically-Independent Reaction Sets

As commented briefly before, one interesting result from the vesicle reactor time evolution Equation (7) is that, in low buffer conditions, two (or more) chemically-independent reaction sets that share the variable vesicle volume will become indirectly coupled. Although each of the reaction sets have an exclusive set of chemical species, the sets still indirectly influence each other by changing the solvent volume in which all reactions are taking place. To our knowledge, this possibility, which could be coined “osmotic coupling”, has been totally neglected in the protocell literature until now. The often followed route has been to assume a single chemically-connected reaction system to constitute a vesicle proto-metabolism.  Nevertheless, osmotic coupling appears as a relevant principle, considering that (i) lipid vesicles are extremely sensitive to osmotic pressure [41,42], like modern cells still are [43,44], and that (ii) in an origins of life scenario, they would constitute micro-environments to carry out “natural experiments” of “combinatorial chemistry” [1] (p. 217), self-assembling in solutions containing many reaction systems performing different and sometimes unrelated chemical transformations.

**Figure 4:**
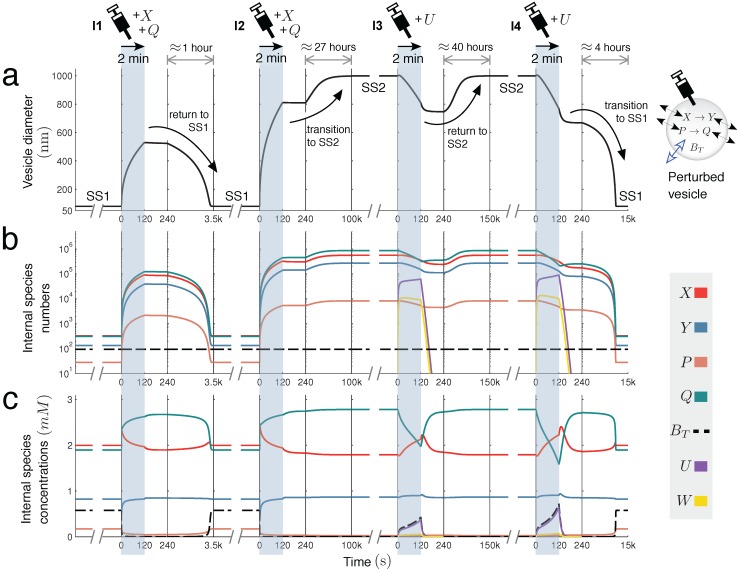
Switching dynamics: bistability in two unimolecular reactions. Encapsulating two unimolecular reactions *X* *→* *Y* and *P* *→* *Q* in the variable-volume vesicle reactor model gives a bistable system under the correct parameter regime (Figure 2c(i)). Below, switching dynamics between steady states SS1 and SS2 are demonstrated by injecting molecules into the reactor by a simulated syringe. Following four different two-minute injections of molecules, changes in (**a**) spherical vesicle diameter, (**b**) vesicle internal species numbers and (**c**) vesicle internal species concentrations are monitored. Injection **I1 **releases both *X* and *Q* into the vesicle at a linear rate of 1000 molecules per second. This perturbation is not sufficiently strong to switch the reactor into SS2, but injection **I2**, releasing *X* and *Q* at 3500 molecules per second, is able to prompt the transition. Once in the larger vesicle SS2 state, the switch back to SS1 is achieved by injecting a new species *U* into the reactor. This species undergoes reaction $U+Q\overset{k}{\to }W$, which depletes *Q* inside the vesicle by quickly transforming it into waste *W* (*k* = 60*.*0) that rapidly diffuses out of the compartment ($D_{W}^{\times }\;=\;100.0,\;D_{U}^{\times }\;=\;1.0$). Injection **I3** releases *U* into the vesicle at a rate of 8000 molecules per second, but cannot initiate the switch back to SS1. Injection **I4** successfully completes the transition, releasing *U* at a rate of 10*,* 000 molecules per second. Time is divided into windows to accommodate different timescales (from minutes to days).

Figure 2c(i) demonstrates that vesicle bistability can emerge quite unexpectedly in our vesicle reactor model from two chemically-independent unimolecular reactions:

(15) $$\begin{array}{c} X\overset{k_{1}}{\to }Y\\ P\overset{c_{1}}{\to }Q \end{array}$$

when these reactions share the internal volume of the vesicle. Figure 4 further explores this interesting case, showing the time dynamics of switching between SS1 and SS2, prompted by extra molecules being injected into the vesicle reactor by a simulated syringe.

Likewise, a unimolecular reaction with two identical products and a chemically-independent bi-molecular reaction with a single product:

(16) $$\begin{array}{c} X\overset{k_{1}}{\to }2Y\\ 2P\overset{c_{1}}{\to }Q \end{array}$$

also display bistability under certain parameter conditions (Figure 2c(ii)).

It can be proven that the individual reactions composing reaction pair Equations (15) and (16) cannot show bistability under any parameter conditions in the CSTR model, nor in the vesicle reactor model (see [26] and the Supplementary Material), and therefore, bistability can be claimed as an emergent feature of the system when the two reactions are present together in the low buffer vesicle model. Incidentally, bistability would be lost if a vesicle were to relocate itself to a region of high external buffer concentration. In high buffer, non-chemically-coupled reaction systems follow independent dynamics, and the non-linearity in the vesicle reactor model is reduced, largely due to the MAK reaction kinetic terms $r_{i}(\vec{s})$ in Equation (4).

**Figure 5:**
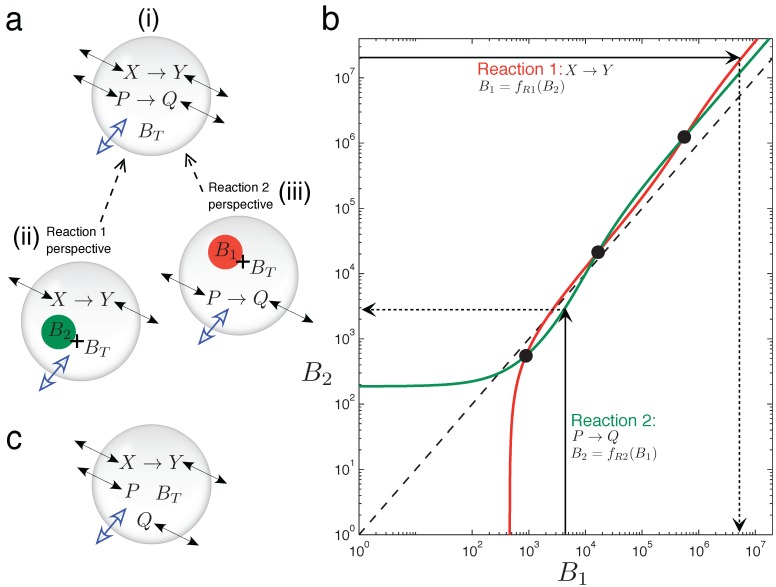
Graphical intuition into emergent bistability through osmotic coupling. （a）Emergent bistability in the vesicle reactor model: (i) two chemically-independent unimolecular reactions can be understood by taking a “reactions-eye view” from the perspective of each reaction; (ii) from the perspective of the *X* *→* *Y* reaction (Reaction 1), all molecules associated with the *P* *→* *Q* reaction simply act as extra inert buffer (*B*_2_) in addition to the trapped impermeable buffer molecules *B_T_* inside the compartment; (iii) conversely, from the perspective of the *P* *→* *Q* reaction (Reaction 2), all molecules associated with the *P* *→* *Q* reaction act as extra inert buffer (*B*_1_). (**b**) Graph showing how the total steady-state particle number of each reaction responds to the extra number of buffer molecules that the other reaction is providing, where Reaction 1 has the y-axis as independent and Reaction 2 has the x-axis as independent. The three cross points represent fulfillment of the cyclic condition referred to in the text. The dotted line shows the relation *B*1 = *B*2. Two chemically-independent reaction sets with identical stoichiometry and identical kinetic constants would give curves that are reflections in this line. (**c**) When the chemical transformation between *P* and *Q* is removed, the latter solutes simply diffuse across the membrane until their respective concentration gradients are equalized. A unimolecular reaction sharing the vesicle compartment with such inert diffusing species cannot be bistable (see the text).

In Figure 5, graphical intuition is developed as to why the two chemically-independent unimolecular reactions, Equation (15), can display emergent bistability in the variable volume vesicle model. It is not at all trivial that two such reactions should display bistability, since both are kinetically simple mechanisms with rates linearly dependent on the reactant concentration. Furthermore, stoichiometrically speaking, there is no net production of molecules in the system (one consumed molecule per produced one, in both reactions), so the asymmetry triggering volume changes can only come from the different diffusion (or permeability) properties of the four chemical species involved.

In order to understand the osmotic coupling, it is convenient to consider the two-reaction system (Figure 5) from the perspective of each reaction. From the view point of the *X* *→* *Y* reaction (Figure 5a(ii)), the species involved in the *P* *→* *Q* reaction cannot have reactive collisions with *X* and *Y* ,and, thus, appear as chemically inert. *P* and *Q* therefore just provide an extra contribution *B*_2_ to the number of impermeable buffer molecules *B*_*T*_ trapped inside the vesicle. Conversely, from the view point of the *P* *→* *Q* reaction (Figure 5a(iii)), species *X* and *Y* appear as inert, and they add *B*_1_ extra buffer molecules to *B_T _*. Therefore, we have the situation that the total number of buffer molecules “experienced” by one reaction depends on the instantaneous species concentrations of the other reaction.

Now, we consider the two reactions to be in the fixed surface vesicle reactor model Equation (9), and analyze how they manage to generate three fixed points (which come from three branches of the bifurcation curve running through the vesicle morphology space).  Figure 5b plots, for a bistable parameter set, how the total particle number that each reaction effectively gives at the steady state is a function of the number of extra buffer molecules that the other reaction is providing [45]. The red line (plotted with the y-axis as the independent variable) is function *B*_1_ = *f_R_*_1_(*B*_2_). This function returns the total effective number of molecules that reaction *X* *→* *Y* (Reaction 1) has at steady state, *B*_1_, given that *B_T_* + *B*_2_ buffer molecules exist inside the vesicle. The green line (plotted normally: x-axis independent) is function *B*_2_ = *f_R_*_2_(*B*_1_), which returns the total effective number of molecules that reaction *P* *→* *Q* (Reaction 2) has at steady state, *B*_2_, given that there are *B_T_* + *B*_1_ buffer molecules inside the vesicle. The whole two-reaction system has a steady state only when the following cyclic condition is fulfilled: Reaction 1, “seeing” *B*_2_ extra buffer molecules inside the vesicle, has a steady state equivalent to *B*_1_ extra buffer molecules, and Reaction 2, “seeing” *B*_1_ extra buffer molecules inside the vesicle, has a steady state equivalent to *B*_2_ extra buffer molecules. The cyclic condition is fulfilled at three points, marked by circles in Figure 5b.

In summary, the potential for bistability in this system comes from the non-linearity of functions *f_R_*_1_ and *f_R_*_2_, *i.e*., from the non-linear response that the steady state of a reaction has to a modification in the total number of buffer molecules inside the vesicle [46]. This non-linearity allows multiple cross points of the red and green curves in Figure 5b. Indeed, writing function *f_R_*_1_ in explicit form (derivation and constants *K* in the Supplementary Material):

(17) $$B_{1}=f_{R1}(B_{2})=(B_{T}\;+B_{2})\frac{K_{1}-K_{4}x^{*}}{K_{3}-K_{4}x^{*}}$$

where the steady-state concentration of species *X* is:

(18) $$x^{*}=\frac{-\left\{K_{3}-x_{\varepsilon }K_{4}+(B_{T}\;+B_{2})\frac{k}{S_{µ}D_{X}}\;\right\}\pm \sqrt{{\left\{K_{3}-x_{\varepsilon }K_{4}+(B_{T}\;+B_{2})\frac{k}{S_{µ}D_{X}}\right\}}^{2}+4K_{4}K_{3}x_{\varepsilon }}}{2K_{4}}$$

we observe that *B*_1_ is a highly non-linear function of *B*_2_. Likewise, *B*_2_ will be a similar non-linear function of *B*_1_.

An important note is that Figure 5b is drawn for a constant vesicle surface area, *i.e.*, for a single horizontal line through vesicle morphology space. Changing the vesicle surface area to generate the bifurcation diagram in morphology space would be represented on Figure 5b as a family of red and green curves. Bifurcation points would be marked as the various crossings of these curves.

Figure 5c shows a curious side case to be considered, in which the chemical transformation involved in reaction *P* *→* *Q* no longer occurs, leaving two inert solutes that just passively diffuse across the vesicle membrane. In this scenario, the whole system loses the potential for bistability. In general, inert diffusing solutes do not change the potential for different steady states in the vesicle reactor model, for each one is restricted to always equilibrate its respective inside-outside gradient. As such, any steady state will see all inert diffusing solutes at the same concentration inside the vesicle as outside (regardless of the diffusion constants of these solutes, the size of the membrane or the volume of the aqueous vesicle water pool). Therefore, in the example of Figure 5c, solutes *P* and *Q* can only change the steady state of the *X* *→* *Y* reaction, in so far as effectively decreasing the total external concentration parameter to *C_ε_−* *p_ε_−* *q_ε_*. Nevertheless, even if inert diffusing solutes cannot expand steady-state possibilities already present, they do change dynamic trajectories toward existing steady states.

## 4. Discussion

### 4.1. Significance of the Results Obtained

Most theoretical modeling and experimental work on (bio-)chemical systems to date has been circumscribed to dilute, well-stirred, fixed-volume conditions in which the system state variables can be restricted to concentrations (or number of molecules) of the main reacting species in aqueous solution. However, this approximation, which incidentally turns out to be a clever and accurate simplification in many domains, may have misguided substantial parts of the research carried out so far about the chemical processes that sustain biological phenomena. The increasing awareness about the problem of “molecular crowding”, for instance, is posing a serious set of challenges to traditional practices in biochemistry [47,48]. The influence of lipid compartments and their dynamic properties on reactive processes taking place around or within them has probably been underestimated, as well. Recent findings on the role that soft interfaces might play to enhance or catalyze the yields of chemical reactions that would be unfavorable in open water solution [49,50,51] give additional support to this thesis. On similar lines, the “fixed-volume assumption” requires careful reconsideration, given the continuous processes of growth, fission and fusion in which biological membranes are typically involved [52].

If we translate the discussion to the context of the origins of life and in particular to research being carried out on protocellular systems (the main target area of the present article and special issue), perhaps the problem of molecular crowding could be set aside temporarily, as far as the initial stages of the vesicle-to-protocell transition are concerned. However, the osmotic movement of water through the semi-permeable membranes of prebiotic vesicles ought to be taken into account right from the very beginning. Variations in the internal volume of these highly flexible and dynamic supramolecular compartments [41] would surely have an important effect on the chemistries taking place within their boundaries, an effect that cannot be simply disregarded.

The theoretical approach and main results here reported constitute a contribution in this generally overlooked direction, a proof of principle to demonstrate that broadening our current experimental and descriptive frameworks for chemical systems to include volume-changing conditions is bound to provide plenty of room for new, unexpected phenomena and insights.

First, we showed that the dynamics of model vesicle compartments can strongly constrain the chemistry within them and may, in fact, degenerate interesting behavior that would otherwise take place in bulk conditions (Case Study 1). However, then, we also showed several other examples (Case Studies 2 and 3) in which the opposite occurs: under the correct parameter regimes, chemistry that is “dull” or trivial in homogeneous free aqueous solution becomes surprisingly interesting when encapsulated in a volume-varying compartment. Even reaction systems that have no chemical species in common (as in Case Study 3) become indirectly coupled to each other when they share the volume where they are realized, an effect that we have coined osmotic coupling. This coupling is mediated by the volume of the “collision space” shared by the reactions (*i.e.*, the solvent) changing via water osmosis. The most striking example of osmotic coupling that we gave was that of two chemically-independent unimolecular reactions, which could lead to a bistable system in the changing volume conditions of the vesicle reactor (Figure 4), provided that the transmembrane diffusion properties of the various molecules involved are different. Alone, either in the CSTR model or the vesicle model, these reactions were incapable of bistability.

### 4.2. Notions of Bistability

When a set of reactions takes place in bulk solution conditions and the mixture is isothermal, from a chemical kinetics point of view, the state of the system is characterized by the instantaneous concentration values of a given number of chemical species. Thus, bistability for this type of classical chemical system (see [53] for a library of examples) refers to the possibility of possessing two unique concentration states that are stable: usually a steady state of zero or very low concentrations and a steady state of high concentrations, often referred to as the “non-trivial” steady state.

In the protocell or vesicle model we introduced here, the notion of bistability is wider. Two stable states can be distinguished not only by the concentrations of the internal solutes, but also by the size of the spherical vesicle that encapsulates them. Figure 2 makes clear that the two stable states for all reaction schemes tested inside the vesicle, SS1 and SS2, generally occur with SS1 at a small SUV vesicle size and SS2 at a larger GUV vesicle size.

In fact, one could say that the size of the vesicle compartment becomes the main factor distinguishing steady states, because the internal solute concentrations are within the same value ranges at both SS1 and SS2. Figure 3 shows that rather than there being “low” and “high” solute concentrations marking steady states, the vesicle re-sizes and the internal concentrations change, but in most cases, only slightly. This should actually be expected, because the osmotic balance condition (2) enforces that the total internal concentration of solutes always be equal to the total external concentration *C_ε_* (fixed). It follows, then, that all reactions inside the vesicle are running at around the same speed (chemical transformations per unit time) in each steady state, which is in contrast to bistable chemical systems in bulk, where, usually, some/all reactions in the “high” state are running at a much accelerated rate. In any case, the absolute number of solute molecules inside the vesicle in SS1 (small vesicle size) is several orders of magnitude less than the number of molecules at SS2 (large vesicle size).

### 4.3. Comments on Graphical Solution Method

In this study, a “graphical method” was introduced (Section 2.1) to find approximate solutions to the set of ODEs describing the full vesicle reactor model Equation (7). This was necessary, because the non-linear dependence of membrane surface area on internal solute concentration Equation (3) destroyed the polynomial property of the ODEs. However, rather than being a poor substitute for a method that would exactly solve Equation (7), the graphical method actually turned out to have significant merits.

The first merit was that it makes obvious how the solutions to Equation (7) are related to fixed point branches that run through the vesicle morphology space. Knowledge of this wider context was essential in obtaining bistable parameter sets in the high dimensional parameter space (see the Methods). For example, whereas an exact solution to Equation (7) may reveal that only one fixed point exists, the graphical method may give the extra information that the underlying bifurcation curve has in fact three branches (only one of which is currently crossing the Φ = 1 line) and that slightly changing parameters would cause three crossings of the Φ = 1 line (and three solutions to the full model). Therefore, the graphical method allows evaluation of how close a parameter set is to giving bistability in the full model, in contrast to an exact solution method, which would only report if bistability exists or not, with no explanation.

The second merit of the graphical method was that it makes explicit all of the possible vesicle shapes (*i.e.*, {Ω*, S_µ_*} pairs in vesicle morphology space) supporting steady-state concentrations of the internal reaction network. In this work, the kinetics of the vesicle surface area were modeled, keeping the restriction of spherical shape: all {Ω*, S_µ_*} pairs on the Φ = 1 line. However, different membrane kinetics schemes may allow wider movement within vesicle viability space, for example if the vesicle surface area is an extra state variable not directly determined by the volume. If this is the case, information on non-spherical {Ω*, S_µ_*}pairs giving a steady state is useful. A visual idea of non-spherical vesicle morphologies supporting a steady state for encapsulated reaction scheme *X* *→* *Y,* *P* *→* *Q* is given in Figure S2 of the Supplementary Material, which represents Points A–F on the bifurcation diagram of Figure 2c(i) as prolate spheroids.

### 4.4. Limitations of the Current Approach

A number of important simplifications have been made in this preliminary approach. We assumed solute diffusion rates across the lipid bilayer to be constant (*i.e.*, not affected by the osmotic tension, shape, curvature or absolute size of the membrane, amongst other factors) and made the assumption that water diffuses instantaneously (when it is just various orders of magnitude faster than for any other species [34]). We also considered the membrane to have negligible thickness, so the properties of the inner and outer bilayer leaflets remain symmetric (e.g., to have equal absorption area *S_µ_*). Besides, we assumed a well-mixed homogeneous medium inside and outside the vesicle reactor, and we followed a deterministic treatment.

A further limitation of our current approach was the “always spherical” membrane kinetics imposed on the system. This can potentially lead to an unrealistic scenario when sharp perturbations are applied to internal or external solute concentrations of the vesicle reactor, causing sudden volume changes. Whereas the model reactor can survive in principle any size perturbation and remain spherical, in reality, lipid monomers from the surrounding solution cannot instantly be absorbed by or leave the membrane to follow volume changes, especially if critical vesicle concentration (CVC) values (*i.e.*, lipid monomer equilibrium concentrations) are very low. In those conditions, a sudden dilution of the environment, for example, will tend to break vesicles as the water volume forces the system to grow much more quickly than what the lipid surface area can accommodate. Figure 4 purposefully uses gradual syringe injections to switch the reactor between steady states, in order to keep the model in realistic operating conditions.

### 4.5. Future Challenges

An immediate extension of the work reported here would be to conduct a more elaborate analysis, using the vesicle reactor model to investigate the osmotic coupling of pairs or triplets of chemically-independent reaction sets that have more species and/or more reactions. For this task, the Supplementary Material details two criteria that could be used to check abstract sets of elementary reactions for physical validity, on the grounds of mass conservation and free energy considerations. Is multi-stability a possibility if the internal reaction sets increase in number and/or complexity? Multi-stable vesicle systems could have the ability to show memory or history dependence to past environmental conditions. Another interesting question is whether different types of emergent chemical behavior, like asymptotically-stable oscillatory limit cycles, could result from osmotically-coupled reaction systems inside the vesicle. If so, what would be the minimal encapsulated reaction system able to trigger and maintain oscillations (which should be reflected both in concentration profiles and vesicle size kinetics)? Detecting oscillations is generally more difficult than detecting bistability, since the former requires extensive dynamics simulation (and re-inclusion of the dilution term Equation (8) in the model), whereas the latter can be inferred just from the existence of fixed points in the model. A technical challenge associated with increasing the degrees of freedom of the model is the numerical algorithm required to reach solutions: we found that the homotopy continuation method to solve Equation (9) noticeably slowed down above four state variables.

Another line of analysis of potential interest (for comparative purposes, as a control of the current approach) would be to relax the immediate flow of water assumption and give water molecules a high (but finite) permeation rate through the bilayer membrane. Vesicle volume would then become an extra degree of freedom in the model. Even if the finite membrane water flow would not affect the number and locations of fixed points in the model phase space (remember, the total internal and external solute concentrations are always equilibrated at fixed points, regardless of how this equilibration is achieved), it could affect their local stability and could lead to different global features of the phase space trajectories. This analysis would test the validity of the assumption expressed through Equation (2).

The osmotic coupling idea from this work might inspire new vesicle experiments measuring the extent to which one set of reactions can skew the dynamics of another chemically-unrelated reaction set, when both proceed inside the same vesicle. Complex reaction sets inside vesicle compartments are already under empirical investigation, like the Belousov–Zhabotinsky reactions in giant phospholipid vesicles [54]. Nevertheless, the idea would be to try simpler chemistries that, in the context of self-assembling micro-compartments, could give rise to unexpected behaviors.

We also expect to relax the constant surface area (Φ = 1) condition and incorporate explicit membrane lipid exchange kinetics (e.g., [9]), which are not directly determined by volume changes, into our theoretical approach to make it more realistic and informative for “*in vitro*” implementations. Finally, as a major and longer term challenge of our line of research, we aim at developing a better understanding of the interaction between proto-metabolic reaction networks and membrane dynamics in this prebiotic, systemic chemistry context.

## 5. Methods

### 5.1. Vesicle Model Parameter Space: Search Methodology

The parameter space for the vesicle model is potentially vast with *d* = (2*N* + *R*_(_*_→_*_)_ + 2*R*_（__⇌__）_ + 2) dimensions [55], where *N* is the number of membrane permeable solute species that are involved in a total of *R*_(_*_→_*_)_ irreversible and *R*_（__⇌__）_ reversible reactions inside the vesicle.

To find parameter regimes in this high-dimensional space that gave at least three branches of the bifurcation curve over vesicle viability space (and, thus, crossing the Φ = 1 line), the following two-phase procedure was followed.

Phase 1, bistability at a constant surface area: The first task was to detect three fixed points (bistability) in a vesicle reactor with a constant surface area. Vesicle surface area $S_{\mu }^{\emptyset }$ was set to that of a 400-nm diameter sphere, and 2000 parameter sets were randomly constructed to perform a very sparse “wide area” Monte-Carlo sampling of the parameter space.

A homotopy continuation algorithm specialized for use with polynomial systems [37,56] was used to solve all isolated fixed points of Equation Set (9) for each parameter set. Homotopy continuation is a powerful and generally convergent numerical method for finding zeros of non-linear functions. The principle (see [57], Chapter 5, for a pedagogical review) is to start with an easily solved problem and then to successively deform this problem, through a series of intermediate problems, into the harder to solve target problem, whilst always tracking the path of the function zeros. Such a method is preferable to solving fixed points by simply simulating and observing the vesicle reactor model. The latter approach suffers from: (i) the necessity to start from many different initial conditions (time consuming); （ii）very slow convergence to some fixed points from certain initial conditions: and, notably, (iii) the inability to find unstable fixed points. Homotopy continuation provided a fast way to sample parameter space accurately.

For the wide area search, parameters were assigned randomly and uniformly in the following ranges. Forward reaction rate constants were assigned *k,c* *∈* (0*,* 10), regardless of the reaction order. Reverse rate constants, if present, were limited to a maximum value, which was a factor of 10 less than the corresponding forward rate constant, *i.e.*, $k_{i}^{r}\in \left(0,\frac{k_{i}}{10}\right)$. Diffusion rates were set from one fifth the permeability of ribose through an oleic acid bilayer, to 50-times that permeability: $D_{i}^{\times }\in \left(\frac{1}{5},50\right)$. This initial range was based approximately on the permeability of sugars and sugar alcohols through oleic acid membranes [34]. External concentrations were assigned in the range $s_{i}^{\varepsilon }\in (0,0.2)$M. The external buffer concentration was assigned in the range 0*.*2 M±40%. Similarly, the number of buffer particles inside a vesicle was assigned 63,064±40%.

Existing fixed point solutions of each parameter set had to pass the following validity checks. The species concentrations at each fixed point were required: (i) to have positive real values; and (ii) to lead to a positive vesicle volume (*i.e.*, $C_{\varepsilon }>\sum_{j=1}^{N}s_{j}^{*}$), which (iii) was not unsuitably outside the size range of GUV vesicles, *i.e.*, Ω*^∗^<* 2000*−*nm diameter sphere. It should be noted that fixed points could have existed outside this maximum volume limit, and they would have been screened out.

In the wide area search, parameter schemes giving bistability in the constant surface reactor Equation (9) often constituted only a very small percentage of all parameter schemes. To amplify the number of bistable candidates, a second stage was performed. In the second stage, a parameter set found to be bistable was used as the basis for a local Monte-Carlo sampling of parameter space. One thousand parameter sets were constructed as variations of the original basis set, whereby all parameters were randomly perturbed ±20% each side of the basis values, using a uniform distribution. This biased sampling of a “hot spot” in parameter space typically resulted in a significantly higher proportion of bistable parameter regimes for manipulation in Phase 2 below.

Phase 2, bistability in vesicle viability space: Section 2.1 explained the algorithm used to graph a bifurcation curve through vesicle morphology space, given a certain parameter set for the vesicle model. After completing Phase 1 above, we knew that its bifurcation curve would contain three distinct fixed points on the horizontal line where the surface area of the vesicle $S_{\mu }^{\emptyset }$ was that of a 400-nm diameter sphere. The bifurcation diagram was assured to therefore have three branches running through the vesicle morphology space at one point at least.

The problem remained as to how to manipulate the bifurcation curve, such that these three branches intersected the vesicle viability region at distinct points within the range of unilamellar vesicle sizes (50-nm diameter to 1500-nm diameter). The shape of the bifurcation curve was often a highly non-linear and unpredictable function of the model parameters. A trial-and-error approach where parameters were randomly varied, followed by immediate re-computation of the bifurcation curve, proved to be a computationally expensive and insufficiently-organized approach.

The problem was solved by realizing that an existing bifurcation curve can be transformed (stretched and translated) to new locations in vesicle morphology space, by scaling some of the model parameters in a structured way. Essentially, even if the form of a bifurcation curve cannot be understood, how to transform an existing curve can be understood.

We can note that each of the *N* multivariate polynomials describing the fixed point solutions of the constant surface vesicle model Equation (9) can be written in the form:

(19) $$\frac{ds_{i}}{dt}=r_{i}(\vec{s})+K_{i}d_{i}(\vec{s})=0\;$$

where **d**_*i*_($\vec{s}$) is the diffusion function for the *i*-th species across the vesicle membrane, and *K_i _* is the constant:

(20) $$K_{i}=S_{µ}\frac{D_{i}}{B_{T}}$$

If the model parameters *S_µ_*_,_ *D_i _*and *B_T_* are changed in such a way that each *Ki* remains the same as previously, then we are solving the same set of multivariate polynomials as before. Hence, the fixed point concentration solutions remain unchanged. However, now, depending on the parameter changes made, the fixed points will happen at shifted {Ω*, S_µ_*} points in morphology space.

To formalize, we introduce new scaling constants *a*, *b*, *c* to write each *K_i _*as:

(21) $$K_{i}=\frac{D_{i}\times a\times c}{B_{T}\times b\times c}\times S_{µ}\left(\frac{b\times c}{a\times c}\right)$$

and observe:

- 1. If each diffusion constant *D_i _*is multiplied by a factor of *a*, then the fixed point volumes stay constant (because *B_T_* remains unchanged and the steady-state concentrations are the same as before), but the fixed point surface areas have to change by a factor of 1*/a* to compensate the scaling of the diffusion constants. The bifurcation curve is stretched vertically up (decreasing *a*) and down (increasing *a*) in vesicle morphology space.
- If each diffusion constant *Di* and the number of trapped buffer molecules *BT* are both multiplied by a common factor *c*, then the fixed point surface areas stay constant (because ratio $\frac{D_{i}}{B_{T}}$ remains the same), but the volume changes by factor *c*, because *B_T_* has been scaled. The bifurcation curve is translated and stretched left (decreasing *c*) and right (increasing *c*) in vesicle morphology space.
- If just *B_T_* is multiplied by a factor *b*, then the surface area and volume of all fixed points are multiplied by *b*. The bifurcation curve is translated and stretched diagonally in vesicle morphology space.

To summarize, each point in the morphology space undergoes the transformation:

(22) $$\left\{Ω,S_{µ}\}\to \{Ω\times bc,S_{µ}\times \frac{b}{a}\right\}$$

By using this transformation in conjunction with some trial-and-error adjustment of parameters, suitable parameter sets from Phase 1 were able to be modified, such that three crossings of the vesicle viability region by the bifurcation branches were obtained (*i.e.*, giving bistability in the full vesicle reactor model Equation (7)). These parameters were then re-scaled into milli-molar concentrations, as detailed below (with *n* = 100), to obtain the bistable parameter regimes underlying Figure 2 (the parameter values themselves are listed in the Supplementary Material).

### 5.2. Rescaling Vesicle Model Parameters for Different Concentration Ranges

The parameters of the fixed surface vesicle model can be changed in a structured way, such that the external and internal steady-state concentrations can be scaled up or down (from molar to millimolar, for example), but yet, with fixed points retaining the same {Ω*, S_µ_*}positions in morphology space. The procedure outlined below leaves the time scale of the dynamics unaffected.

Suppose the required concentration range for all internal and external solutes, in molar, is a factor of *n* less than the current molar concentration range:

(23) $$s_{i}^{\prime }=\frac{s_{i}}{n}$$

Consider a permeable solute, say *X*, which is consumed by a first-order reaction, a second-order reaction and a third-order reaction inside the vesicle. The concentration derivative for *X* in the fixed surface reactor ODE set Equation (9) reads:

(24) $$\frac{dx}{dt}=-k_{1}x-k_{2}xy-k_{3}xz^{2}\;+\frac{S_{µ}^{\emptyset }D_{X}}{B_{T}}\;\left(C_{\varepsilon }-\sum_{j=1}^{N}s_{j}\right)(x_{\varepsilon }-x)=0$$

By substituting all concentrations with their scaled equivalents *si* = *n*$s_{i}^{\prime }$, Equation (23), we arrive at an expression equivalent to (24) above:

(25) $$-k_{1}(nx^{\prime })-k_{2}(nx^{\prime })(ny^{\prime })-k_{3}(nx^{\prime })(nz^{\prime })(nz^{\prime })+\frac{S_{µ}^{\emptyset }D_{X}}{B_{T}}\left(nC_{\varepsilon }^{\prime }-n\sum_{j=1}^{N}s_{j}^{\prime }\right)(nx_{\varepsilon }^{\prime }-nx^{\prime })=0$$

Factoring by *n*:

(26) $$n\left\{-k_{1}x^{\prime }\;-nk_{2}x^{\prime }y^{\prime }\;-n^{2}k_{3}x^{\prime }{\left(z^{\prime }\right)}^{2}\;+n\frac{S_{µ}^{\emptyset }D_{X}}{B_{T}}\left(C_{\varepsilon }^{\prime }-\sum_{j=1}^{N}s_{j}^{\prime }\right)(x_{\varepsilon }^{\prime }-x^{\prime })\right\}=0$$

The expression in the curly bracket shows how the model parameters must absorb the scaling factors *n*, in order to give a zero at the scaled concentrations. First order reaction rates (e.g., *k*_1_) remain unchanged; second order reaction rates (e.g., *k*_2_) require multiplying by *n* and third order reaction rates (e.g., *k*_3_) multiplying by *n*^2^. Dividing the number of trapped internal buffer particles *B_T_* by *n* ensures that the scaled concentrations create the same steady-state aqueous volume of the system Ω as before, *i.e.*:

(27) $$Ω=\frac{B_{T}/n}{(C_{\varepsilon }-\sum_{j=1}^{N}s_{j}\;)/n}$$

and this operation also has the effect of multiplying term $\frac{S_{\mu }^{\emptyset }D_{x}}{B_{T}}$ by *n*, as required. The surface $S_{\mu }^{\emptyset }$ of the system stays the same as before, as do the diffusion constants *D_X _*.

### 5.3. Vesicle Parameter Set Ranges For Schlögl and Wilhelm Models: Case Study 1

Parameters for the fixed surface vesicle model were assigned randomly and uniformly in the following ranges: diffusion constants, $D_{i}^{\times }\in (\frac{1}{5},50)$; external solute concentrations, except for those corresponding to reservoir species in the original models, $s_{i}^{\varepsilon }$ *∈* (0*,* 0*.*002) M; external buffer concentration, 0.002 M ± 40%; the number of buffer particles trapped inside the vesicle, *B_T_∈* (2*,* 2000).

## Supplementary Materials

Supplementary materials can be accessed at: http://www.mdpi.com/2075-1729/5/1/181/s1.
