# Supplementary material for: Emergent Chemical Behavior in Variable-Volume Protocells
Source: Life (Basel). 2015 Jan 13;5(1):181–211. doi: 10.3390/life5010181 (PMC4390847; doi:10.3390/life5010181)
Supplement: Supplementary file 1 [file life-05-00181-s001.pdf]

Supplementary Materials

1. Equivalent Concentration and Particle Formulations of Vesicle Solute Dynamics

A well-stirred chemical reaction system is traditionally formalised as a set of deterministic concentration ODEs. Figure 1 makes clear the relationship of this traditional approach, to less traditional scenarios where there are still concentration ODEs but the solvent volume is changing, or where the evolution of absolute particle numbers is focussed on instead.

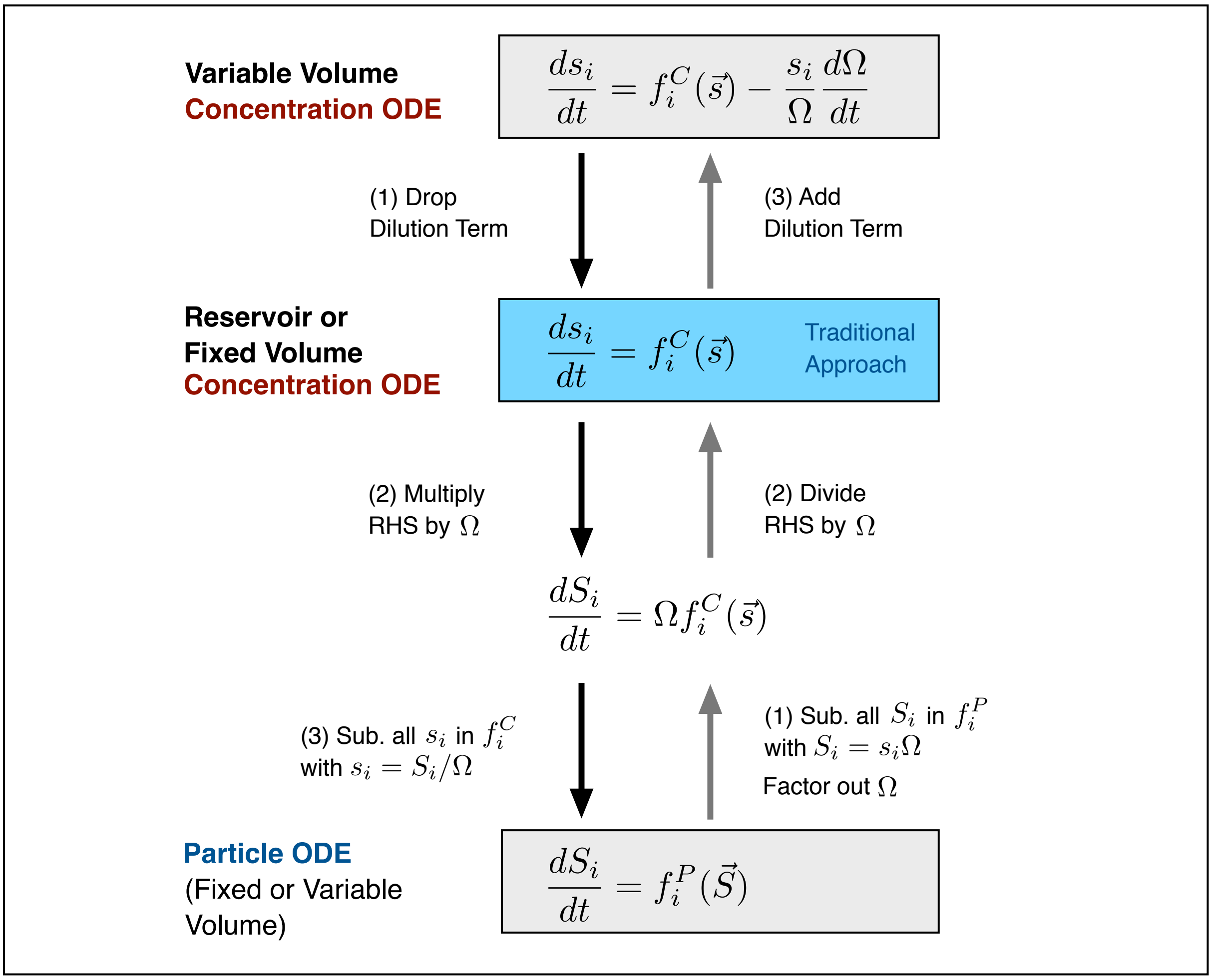

Figure 1. Relationship between Traditional Concentration ODE, Variable Volume Concentration ODE and Particle ODE Formalisms. The traditional approach to chemical kinetics has been to explore reactions in fixed or reservoir solvent volumes (blue box). This approach can be related to formalisms dealing with finite and variable solvent volumes (grey boxes). Notation: Function f_i^C returns the rate of concentration change of the i'th solute, based on current solute concentrations $\vec{s}$. Function f_i^P returns the rate of particle number change of the i'th solute, based on current solute particle numbers $\vec{S}$.

It is useful to bear in mind:

- **Concentration ODEs** describe the time evolution of solute *number densities* in a volume. For our vesicle model, the **three** factors directly affecting the number density of solutes inside the

vesicle are (i) chemical reactions, (ii) diffusions into/out of the volume, and (ii) changes in vesicle volume itself.

- **Particle ODEs** describe the time evolution of solute *particle numbers* in a volume. For our vesicle model, the **two** factors directly affecting absolute particle numbers inside the vesicle are (i) chemical reactions and (ii) diffusions into/out of the volume. Changing the vesicle solvent volume itself does **not directly** affect the number of solute particles. Rather, it changes the particle reaction rate constants (which in turn affect the particle numbers in future).

For our vesicle reactor model, the variable volume concentration ODE formalism is generally much easier to solve for steady states (analytically, or numerically) than is the particle ODE representation, because it is always a set of multivariate polynomials in the species concentrations. The particle ODE set is generally unattractive to solve for steady state particle numbers, since it can contain rational fractions.

For numerically integrating the dynamics of the vesicle reactor model via Runge-Kutta, the particle ODE was found to be the most stable and reliable approach. This is because, under the particle formalism, the volume of the vesicle changes linearly with the number of particles, regardless of vesicle size. Conversely, under the concentration formalism, the vesicle volume is the (non-linear) reciprocal of $C_{\mathcal{E}} - \sum_{j=1}^{N} s_j$, which means that as the vesicle volume grows larger, the total internal solute concentration $\sum_{j=1}^{N} s_j$ approaches the total external concentration $C_{\mathcal{E}}$, and here only exceedingly small concentration changes cause very large fluctuations in volume. Generally, the particle and concentration formalisms were identical up to vesicle diameters of d=1000nm, after which notable numerical integration errors were present in the concentration approach.

A final note is that the particle ODE is sometimes impractical to use for systems which have large volume because the numbers of particles involved become very large indeed and impractical to handle on a computer.

1.1. Example: Schlögl Model in Vesicle Reactor

In the following example, the ODE set for the bistable Schlögl reaction set

$$X + 2Y \underset{k_1^r}{\overset{k_1}{\rightleftharpoons}} 3Y$$

$$Y \underset{k_2^r}{\overset{k_2}{\rightleftharpoons}} Z$$

in the vesicle reactor model is written in both concentration and particle representations. The vesicle reactor is first assumed to have fixed volume $\Omega^{\oslash}$. The (traditional) concentration ODEs read:

$$\frac{dx}{dt} = -k_1 x y^2 + k_1^r y^3 + \frac{1}{\Omega^{\oslash}} S_\mu D_X (x^{\mathcal{E}} - x)$$

$$\frac{dy}{dt} = k_1 x y^2 - k_1^r y^3 - k_2 y + k_2^r z + \frac{1}{\Omega^{\oslash}} S_\mu D_Y (y^{\mathcal{E}} - y)$$

$$\frac{dz}{dt} = k_2 y - k_2^r z + \frac{1}{\Omega^{\oslash}} S_\mu D_Z (z^{\mathcal{E}} - z)$$

and the equivalent particle ODEs read [1]:

$$\frac{dX}{dt} = -\frac{k_1}{(\Omega^{\oslash})^2}XY^2 + \frac{k_1^r}{(\Omega^{\oslash})^2}Y^3 + S_\mu D_X(x^{\mathcal{E}} - \frac{X}{\Omega^{\oslash}})$$

$$\frac{dY}{dt} = \frac{k_1}{(\Omega^{\oslash})^2}XY^2 - \frac{k_1^r}{(\Omega^{\oslash})^2}Y^3 - k_2Y + k_2^rZ + S_\mu D_Y(y^{\mathcal{E}} - \frac{Y}{\Omega^{\oslash}})$$

$$\frac{dZ}{dt} = k_2Y - k_2^rZ + S_\mu D_Z(z^{\mathcal{E}} - \frac{Z}{\Omega^{\oslash}})$$

When the volume is made to change as a direct function of internal solute concentrations, as in the vesicle reactor model, the above particle ODE set is still valid. The fixed volume $\Omega^{\oslash}$ is simply replaced with function $\Omega = (X + Y + Z + B_T)/C_{\mathcal{E}}$.

However, under changing volume, the traditional concentration ODE set is *no longer valid*. To be valid again, each concentration derivative needs an extra dilution term adding (Figure 1). Only then can the fixed volume $\Omega^{\oslash}$ be legitimately replaced with function $\Omega = B_T/(C_{\mathcal{E}} - x - y - z)$. The section below explains the dilution term in more detail, and how it can be formulated in terms of the instantaneous species concentrations.

2. Dilution Term

Below in Sections 2.1 and 2.2 we derive the dilution term introduced by Pawlowski and Zielenkiewicz [2], to handle reaction systems in a variable solvent volume. Then, in Sections 2.3 and 2.4, we derive what the dilution term must be in a reaction system whose volume is a function of the internal solute concentrations (as in our vesicle reactor model).

2.1. Derivation via Quotient Rule

In well-mixed conditions, the instantaneous concentration of a solute species s_i is defined as the number of molecules of the species present divided by the current solvent volume:

$$s_i(t) = \frac{S_i(t)}{\Omega(t)}$$

We can think of s_i as a function, returning the concentration of species s_i at time t. Likewise, functions $S_i(t)$ and $\Omega(t)$ will return the number of molecules and current volume at time t, respectively. Taking the derivative of function s_i with respect to t is the same as taking the derivative of function fraction S_i/Ω with respect to t, and thus we can apply the Quotient Rule from Calculus:

$$\frac{ds_i}{dt} = \frac{d}{dt}(S_i/\Omega) = \left(\frac{\Omega\frac{dS_i}{dt} - S_i\frac{d\Omega}{dt}}{\Omega^2}\right) = \left(\frac{1}{\Omega}\frac{dS_i}{dt} - \frac{S_i}{\Omega^2}\frac{d\Omega}{dt}\right) = \left(\frac{1}{\Omega}\frac{dS_i}{dt} - \frac{s_i\Omega}{\Omega^2}\frac{d\Omega}{dt}\right)$$

Giving the final result:

$$\frac{ds_i}{dt} = \frac{1}{\Omega}\frac{dS_i}{dt} - \frac{s_i}{\Omega}\frac{d\Omega}{dt}$$

This equation states that, in a variable volume, the rate of change of species s_i is equal to the rate of change we would expect in a fixed volume, *i.e.*,

$$\frac{ds_i}{dt} = \frac{1}{\Omega}\frac{dS_i}{dt}$$

minus a dilution term to compensate for the volume changing. If the volume is decreasing ($\frac{d\Omega}{dt} < 0$) the dilution term becomes positive and acts to further increase the concentration of a species (and *vice versa*).

2.2. Alternative Derivation

Recall

$$s_i = \frac{S_i}{\Omega}$$

If the volume is changed by an amount $\Delta\Omega$, there will be an associated change Δs_i in the species concentration:

$$s_i + \Delta s_i = \frac{S_i}{\Omega + \Delta\Omega}$$

Assuming no particles escaped or entered the volume whilst we made the volume change, the total number of particles after the change will be the same as before it, *i.e.*, $S_i = s_i\Omega$:

$$s_i + \Delta s_i = \frac{s_i\Omega}{\Omega + \Delta\Omega}$$

Expanding and re-arranging to isolate the Δs_i term gives:

$$s_i\Omega = (s_i + \Delta s_i)(\Omega + \Delta\Omega)$$

$$s_i\Omega = s_i\Omega + \Delta s_i\Omega + s_i\Delta\Omega + \Delta s_i\Delta\Omega$$

$$0 = \Delta s_i(\Omega + \Delta\Omega) + s_i\Delta\Omega$$

$$\Delta s_i = -s_i\frac{\Delta\Omega}{\Omega + \Delta\Omega}$$

where the minus sign expresses that concentration s_i decreases as the volume increases, as expected. If the volume change is *infinitesimal*, then the denominator $\Omega + d\Omega \approx \Omega$ and thus the infinitesimal change in species concentration is:

$$ds_i = -s_i\frac{d\Omega}{\Omega} = -\frac{s_i}{\Omega}d\Omega$$

which is another statement of the dilution term. To recap, it says that if of the solvent volume changes by $d\Omega$, with all other things being equal, the concentration of solute originally at s_i will change to $s_i - \frac{s_i}{\Omega}d\Omega$.

2.3. Dilution Term for Vesicle Model: Derivation

In the vesicle reactor model, the system volume is a function of the internal solute concentrations. Here, we derive what the dilution term is in this context.

2.3.1. Step 1

First, consider that the vesicle reactor has a fixed volume. The factors affecting the rate of change of the species concentrations inside this volume are the MAK reaction kinetics functions $\mathbf{r}_j(\vec{s})$ and passive diffusions into and out of the vesicle. For each species s_i:

$$\frac{ds_i}{dt} = \mathbf{r}_i(\vec{s}) + \frac{1}{\Omega^{\oslash}} S_\mu D_i(s_i^{\mathcal{E}} - s_i) \tag{1}$$

where $\Omega^{\oslash}$ is the fixed volume parameter.

2.3.2. Step 2

Next, the derivatives (1) are multiplied by the current volume Ω (see Figure 1 above) so that they describe the rate of change of species molecule numbers or 'particles' inside the vesicle, in terms of the current species concentrations:

$$\frac{dS_i}{dt} = \Omega \mathbf{r}_i(\vec{s}) + S_\mu D_i(s_i^{\mathcal{E}} - s_i) \tag{2}$$

By describing rates of change of particle numbers, these derivatives are valid when the volume becomes variable. Conversely, concentration derivatives (1) above are not valid when the volume becomes variable.

2.3.3. Step 3

Using the vesicle volume specified in terms of particles (Figure 1 above), we can easily write how a change in the vesicle volume is related to a change in the total internal particle number:

$$\Delta\Omega = \left(\frac{B_T + (S_1 + \Delta S_1) + \cdots + (S_N + \Delta S_N)}{C_{\mathcal{E}}}\right) - \left(\frac{B_T + S_1 + \cdots + S_N}{C_{\mathcal{E}}}\right)$$

Giving

$$\Delta\Omega = \frac{1}{C_{\mathcal{E}}} \sum_{j=1}^{N} \Delta S_j$$

where ΔS_j is a change in the copy number of the jth species. This relationship is valid for any changes in the number of internal particles, including infinitesimal changes:

$$d\Omega = \frac{1}{C_{\mathcal{E}}} \sum_{j=1}^{N} dS_j$$

If infinitesimal changes are related as such, then their rates of change in time are similarly related:

$$\frac{d\Omega}{dt} = \frac{1}{C_{\mathcal{E}}} \sum_{j=1}^{N} \frac{dS_j}{dt} \tag{3}$$

Therefore, the vesicle volume derivative is simply a scaled sum of the solute particle derivatives.

2.3.4. Step 4

Equation (2) above gives the solute particle derivatives in terms of the instantaneous solute concentrations. Therefore, substituting (2) into (3) gives the volume derivative in terms of instantaneous concentrations:

$$\frac{d\Omega}{dt} = \frac{1}{C_{\mathcal{E}}} \sum_{j=1}^{N} \left(\Omega \mathbf{r}_j(\vec{s}) + S_\mu D_j(s_j^{\mathcal{E}} - s_j)\right)$$

Alternatively:

$$\frac{d\Omega}{dt} = \frac{\Omega}{C_{\mathcal{E}}} \sum_{j=1}^{N} \left(\mathbf{r}_j(\vec{s}) + \frac{1}{\Omega} S_\mu D_j(s_j^{\mathcal{E}} - s_j)\right) \tag{4}$$

2.3.5. Step 5

The full dilution term, including the volume derivative, is thus:

$$-\frac{s_i}{\Omega}\frac{d\Omega}{dt} = -\frac{s_i}{C_{\mathcal{E}}} \sum_{j=1}^{N} \left(\mathbf{r}_j(\vec{s}) + \frac{1}{\Omega} S_\mu D_j(s_j^{\mathcal{E}} - s_j)\right)$$

In other words, the dilution term for solute s_i is simply the sum of all terms in the N concentration ODEs for the fixed volume system, multiplied by $-s_i/C_{\mathcal{E}}$. Note that $\sum_{j=1}^{N} \mathbf{r}_j(\vec{s})$ has a nett value of 0 when each reaction produces the same number of molecules that it consumes.

Substituting the volume $\Omega = B_T/(C_{\mathcal{E}} - \sum_{j=1}^{N} s_j)$, makes it clear that the dilution term is a multivariate polynomial in the species concentrations.

2.4. Dilution Term for Vesicle Model: Alternative Derivation

The total concentration of solutes inside the vesicle is equal to the total concentration of solutes outside, when the isotonic condition holds at all times:

$$C_{\mathcal{C}} = C_{\mathcal{E}}$$

The volume change of the vesicle ensures that the **total** concentration of solutes inside the vesicle does not change, despite reactions and diffusions across the membrane changing the concentrations of the **individual** solute species. Following Mavelli and Ruiz-Mirazo[3], we can write:

$$\frac{dC_{\mathcal{C}}}{dt} = 0 = \sum_{j=1}^{N} \left(\mathbf{r}_j(\vec{s}) + \frac{1}{\Omega} S_\mu D_j(s_j^{\mathcal{E}} - s_j)\right) - \frac{C_{\mathcal{C}}}{\Omega}\frac{d\Omega}{dt}$$

Which gives the volume derivative of the vesicle as:

$$\frac{d\Omega}{dt} = \frac{\Omega}{C_{\mathcal{C}}} \sum_{j=1}^{N} \left(\mathbf{r}_j(\vec{s}) + \frac{1}{\Omega} S_\mu D_j (s_j^{\mathcal{E}} - s_j) \right) \tag{5}$$

The same as Equation (4), taking into account that $C_{\mathcal{C}} = C_{\mathcal{E}}$.

3. Physical Constraints on Abstract Chemical Reaction Sets

Often in artificial "toy" chemistries, reactions are specified at an abstract level, for example $A + B \rightarrow C$. Under this notation, the atomic structure and physical state of a molecule is reduced to a single letter. Despite this brutal abstraction, sets of such reactions can still be verified as physically plausible to some extent.

Below are described two constraints, which can be applied together, if all reactions (i) are elementary and (ii) describe the transformation of reactant complexes at higher Gibbs free energy to product complexes at lower Gibbs free energy. These constraints are useful to apply when automatically generating more complicated reaction sets for testing in the vesicle reactor.

3.1. Atom Number Conservation

Traditional *elemental balance* cannot be performed with an artificial chemistry, since the exact atomic constituents of molecules have been abstracted away. However, conservation of the *total atom number* in the system can be verified by rewriting a set of reactions as a set of linear simultaneous equations, where each equation stipulates that the number of atoms on the left and right hand sides has to be conserved. To do this, every reaction arrow (regardless if irreversible, or bi-directional) is re-written as an equals sign, and the species letters become variables signifying the number of atoms in a molecule of that type. If positive non-zero solutions can be found (one or infinitely many), then this means that all species can be assigned an atom number such that no sequence of reactions will neither create nor destroy atoms. Table 1 gives an example of a reaction set conserving atoms, and Table 2 gives three reaction sets which don't. The order of the reaction kinetics do not affect this procedure, since only the change of species numbers on execution of a reaction are relevant, and not reaction rates.

Table 1. A reaction set which passes atom number conservation, checked by solving two simultaneous equations.

Reaction Set	Atom Number Conservation	Simultaneous Equations
$2X \rightleftharpoons Z$	$2X = Z$	$2X - Z = 0$
$2Y \rightarrow X + Z$	$2Y = X + Z$	$2Y - X - Z = 0$
Solutions: $X = \frac{1}{2}Z,\ \ Y = \frac{3}{4}Z$		

Table 2. Three reaction sets failing atom number conservation.

Trivial	Less Obvious	More Subtle
$2X \rightleftharpoons X$	$X \rightarrow Y$	$2Z \rightarrow X + Y$
	$2Y \rightarrow X$	$W \rightarrow X + Z$
		$X + Z \rightarrow Y + W$

3.2. Existence of Free Energy Simplex

Reactions are driven by a thermodynamic tendency to achieve a lower Gibbs energy. For a reaction to proceed spontaneously, the total Gibbs free energy of the reactant species must be higher than the total free energy of the product species. To check this constraint, a reaction set can be transformed into a set of linear inequalities, with each reaction arrow pointing in the spontaneous direction, re-written as a greater-than $>$ symbol. If a set of *all positive* solutions exists to this set of inequalities (*i.e.*, a solution simplex is defined in the positive orthant of N-dimensional space, where N is the number of species), then this means that all reactions can have a negative free energy change, and the reaction set is subsequently valid from a free energy perspective. Table 3 shows the inequalities for a valid reaction set, and Table 4 gives two examples of reaction sets failing the negative free energy test, which include perpetual-motion cycles (incidentally, these pass the atom number conservation criterion above).

Table 3. A reaction set which passes negative free energy test, and atom number conservation.

Reaction Set	Free Energy Inequalities
$X + Z \rightleftharpoons 2Y$	$X + Z > 2Y$
$Y + Z \rightarrow 2X$	$Y + Z > 2X$
	$X > 0$
	$Y > 0$
	$Z > 0$
One possible solution: $X = 1.0, \quad Y = 2.0, \quad Z = 3.1$ (in arbitrary units of Gibbs free energy)	

Table 4. Two reaction sets failing negative free energy criterion. Both reaction sets incidentally pass atom number conservation.

Perpetual cycle with no energy input	Another example
$X \rightarrow Y$	$2X \rightarrow 2Z$
$Y \rightarrow Z$	$Y + Z \rightarrow 2X$
$Z \rightarrow X$	$Z \rightarrow X$

For large reaction sets, the existence of solutions for both of the constraints outlined above can be checked by a computer algebra system, e.g., *MuPad*, *Maple* or *Mathematica*.

4. Non-Bistability Demonstrations

In this section, simple algebra is used to prove that four reactions used in the paper cannot show bistability in different chemical reactor scenarios, regardless of the parameter scheme used.

4.1. Irreversible Reaction Sequence 2X –> Z –> 2Y

4.1.1. Reservoir Conditions

With X designated as high energy resource (fixed concentration), and Y as low energy waste (fixed concentration), intermediate species Z is described by:

$$\frac{dz}{dt} = k_1 x^2 - k_2 z$$

which has only a single steady state solution:

$$z^* = \frac{k_1}{k_2} x^2$$

4.1.2. CSTR Flow Conditions

Species Y is produced as inert waste, by an irreversible reaction. Therefore, its concentration in the CSTR is of no consequence to the reaction, and it can be disregarded. The system reduces to:

$$\frac{dx}{dt} = -2k_1 x^2 + \frac{1}{\theta}(x_f - x)$$

$$\frac{dz}{dt} = k_1 x^2 - k_2 z + \frac{1}{\theta}(z_f - z)$$

Setting the x derivative to 0 gives quadratic

$$(2\theta k_1)x^2 + x - x_f = 0$$

which will have a maximum of two positive steady state solutions for x. For each of these steady state solutions x^*, from setting the z derivative to zero, we see that there exists a single corresponding steady state solution for z:

$$z^* = \frac{\theta k_1 (x^*)^2 + z_f}{\theta k_2 + 1}$$

Therefore, at most, there exists two unique $\{x^*, z^*\}$ pairs. A minimum of three fixed points are required for bistability (two stable fixed points whose basins of attraction are separated by an unstable saddle point), and so this reaction sequence cannot be bistable in CSTR flow conditions.

4.2. Irreversible Reaction Sequence X –> 2Z –> Y

4.2.1. Reservoir Conditions

With X designated as high energy resource (fixed concentration), and Y as low energy waste (fixed concentration), intermediate species Z is described by:

$$\frac{dz}{dt} = 2k_1 x - 2k_2 z^2$$

which has only a single steady state solution, since negative concentrations are not permitted:

$$z^* = +\sqrt{\frac{k_1}{k_2}x}$$

4.2.2. CSTR Flow Conditions

Species Y is produced as inert waste, by an irreversible reaction. Therefore, its concentration in the CSTR is of no consequence to the reaction, and it can be disregarded. The system reduces to:

$$\frac{dx}{dt} = -k_1 x + \frac{1}{\theta}(x_f - x)$$

$$\frac{dz}{dt} = 2k_1 x - 2k_2 z^2 + \frac{1}{\theta}(z_f - z)$$

From setting the x derivative to 0, we see that x admits only one steady state solution:

$$x^* = \frac{x_f}{\theta k_1 + 1}$$

Substituting x^* into the z derivative, gives a quadratic function for z^*, ruling out the possibility for at least three unique $\{x, z\}$ solution pairs:

$$2k_2 z^2 + \frac{1}{\theta}z - \left\{2k_1\left(\frac{x_f}{\theta k_1 + 1}\right) + \frac{1}{\theta}z_f\right\} = 0$$

At most, the system could have one stable, and one unstable fixed point.

4.3. Irreversible First-Order Reaction X –> nY

Constant $n > 0$ describes one or many reaction products.

4.3.1. Reservoir Conditions

This reaction cannot be assessed under reservoir conditions, because there are no intermediate species to dissipate energy between the high and low energy reservoirs.

4.3.2. CSTR Flow Conditions

Species Y is produced as inert waste, by an irreversible reaction. Therefore, its concentration in the CSTR is of no consequence to the reaction, and it can be disregarded. The system reduces to a single derivative:

$$\frac{dx}{dt} = -kx + \frac{1}{\theta}(x_f - x)$$

and there can exist only a single steady state solution when:

$$x^* = \frac{x_f}{\theta k + 1}$$

4.3.3. Vesicle Conditions

At steady state:

$$\frac{dx}{dt} = -kx + \frac{1}{\Omega^*} S_\mu D_X (x_\mathcal{E} - x) = 0 \tag{6}$$

$$\frac{dy}{dt} = nkx + \frac{1}{\Omega^*} S_\mu D_Y (y_\mathcal{E} - y) = 0 \tag{7}$$

Dividing the x steady state expression (6) by the y steady state expression (7) usefully simplifies:

$$\frac{D_X(x_\mathcal{E} - x)}{D_Y(y_\mathcal{E} - y)} = -n \tag{8}$$

and yields the following linear relation between the x and y steady state values:

$$y^* = \left(\frac{D_X}{nD_Y} x_\mathcal{E} + y_\mathcal{E}\right) - \left(\frac{D_X}{nD_Y}\right) x^*$$

$$y^* = K_1 - K_2 x^* \tag{9}$$

At steady state, the reciprocal of vesicle volume becomes:

$$\frac{1}{\Omega^*} = \frac{1}{B_T}(C_\mathcal{E} - x - y) = \frac{1}{B_T}(C_\mathcal{E} - x - K_1 + K_2 x)$$

which substituted into (6) gives a quadratic in x, with at most two positive solutions:

$$\frac{dx}{dt} = -kx + \frac{S_\mu D_X}{B_T}(K_3 + K_4 x)(x_\mathcal{E} - x) = 0 \tag{10}$$

where constants $K_3 = C_\mathcal{E} - K_1$, $K_4 = K_2 - 1$.

Therefore, the $X \rightarrow nY$ reaction cannot achieve bistability in the vesicle model under any parameter conditions.

4.4. Irreversible Bi-molecular Reaction 2X –> Y

4.4.1. Reservoir Conditions

This reaction cannot be assessed under reservoir conditions.

4.4.2. CSTR Flow Conditions

Species Y is produced as inert waste, by an irreversible reaction. Therefore, its concentration in the CSTR is of no consequence to the reaction, and it can be disregarded. The system reduces to a single derivative:

$$\frac{dx}{dt} = -2kx^2 + \frac{1}{\theta}(x_f - x)$$

At steady state, the x concentrations are given by quadratic:

$$2\theta kx^2 + x - x_f = 0$$

and hence a maximum of only two positive solutions can exist (where one will be a stable fixed point, the other an unstable saddle).

4.4.3. Vesicle Conditions

At steady state:

$$\frac{dx}{dt} = -2kx^2 + \frac{1}{\Omega^*}S_\mu D_X(x_\mathcal{E} - x) = 0 \tag{11}$$

$$\frac{dy}{dt} = kx^2 + \frac{1}{\Omega^*}S_\mu D_Y(y_\mathcal{E} - y) = 0 \tag{12}$$

Dividing the x steady state expression (11) by the y steady state expression (12) gives:

$$\frac{D_X(x_\mathcal{E} - x)}{D_Y(y_\mathcal{E} - y)} = -2$$

which is a specific case of condition (8). Following the same logic as above in Section 4.3.3 with $n = 2$, the x derivative at steady state will be:

$$\frac{dx}{dt} = -2kx^2 + \frac{S_\mu D_X}{B_T}(K_3 + K_4 x)(x_\mathcal{E} - x) = 0$$

which still remains quadratic function of x.

5. Response of X –> Y Reaction Steady States to Changing Buffer in Vesicle Reactor

In this section, we calculate for reaction $X \xrightarrow{k} Y$ inside a vesicle (called reaction 1), how the total concentration of X and Y solutes at steady state depends upon the total number of buffer molecules $B_T + B_2$ inside the vesicle. Parameter B_T is used to denote the number of inert and impermeable buffer particles trapped permanently inside the vesicle interior. Parameter B_2 denotes the extra *effective* number of buffer particles, provided by the other reaction in the vesicle, reaction 2.

As already established (10) for reaction 1, at steady state:

$$\frac{dx}{dt} = -kx + \frac{S_\mu D_X}{B_T + B_2}(K_3 + K_4 x)(x_\mathcal{E} - x) = 0$$

That is, fixed points are given by quadratic function

$$x^* = x \mid ax^2 + bx + c = 0 \tag{13}$$

where polynomial coefficients

$$a = K_4$$

$$b = K_3 - x_\mathcal{E} K_4 + (B_T + B_2)\frac{k}{S_\mu D_X}$$

$$c = -K_3 x_\mathcal{E}$$

The related y fixed point was given in Equation (9).

When the reaction concentrations are at a fixed point, the volume of the vesicle is

$$\Omega^* = \frac{B_T + B_2}{C_\mathcal{E} - x^* - y^*} \tag{14}$$

which means that this reaction would be providing

$$B_1 = \Omega^*(x^* + y^*) \tag{15}$$

effective buffer particles to the other reaction (reaction 2) at the fixed points.

To elaborate the full function $B_1 = f_{R1}(B_2)$ to see the complicated dependence of $B1$ on $B2$, first Ω^* (14) is substituted into (15) to give:

$$B_1 = (B_T + B_2)\,\frac{x^* + y^*}{C_\mathcal{E} - x^* - y^*}$$

Substituting the y fixed point concentration (9):

$$B_1 = (B_T + B_2)\,\frac{K_1 - K_4 x^*}{K_3 + K_4 x^*}$$

where x^* is given by the quadratic formula as applied to (13):

$$x^* = \frac{-\left\{K_3 - x_\mathcal{E} K_4 + (B_T + B_2)\frac{k}{S_\mu D_X}\right\} \pm \sqrt{\left\{K_3 - x_\mathcal{E} K_4 + (B_T + B_2)\frac{k}{S_\mu D_X}\right\}^2 + 4K_4 K_3 x_\mathcal{E}}}{2K_4}$$

and so B_1 has a very complicated non-linear dependence on B_2.

6. Parameter and Fixed Point Concentration Tables to Accompany Figure 2 and 3 of Paper

Table 5. Concentration, Diffusion, Reaction and Buffer Parameters for Bistable Vesicles in Figure 2 of paper. All values in scientific notation. External concentrations in M. Membrane diffusion parameters $D_i^{\times}$ specified as multipliers (see paper). First order reaction rate constants in units s^{-1}, second order in M^{-1}s^{-1}, third order in M^{-2}s^{-1}.

	**a(i)**	**a(ii)**	**b(i)**	**b(ii)**	**c(i)**	**c(ii)**
$x_{\mathcal{E}}$	$1.4024e-3$	$7.3135e-4$	$1.8058e-3$	$1.0896e-3$	$2.0160e-3$	$1.1828e-3$
$y_{\mathcal{E}}$	$1.8225e-3$	$1.7705e-3$	$4.1160e-4$	$1.9567e-4$	$8.1789e-4$	$7.9604e-5$
$z_{\mathcal{E}}$	$1.9225e-3$	$1.9779e-3$	$1.6923e-3$	$5.3546e-4$	–	–
$w_{\mathcal{E}}$	–	$1.1944e-3$	–	–	–	–
$p_{\mathcal{E}}$	–	–	–	–	$3.3171e-4$	$2.2131e-3$
$q_{\mathcal{E}}$	–	–	–	–	$9.2989e-4$	$1.8946e-3$
$b_{\mathcal{E}}$	$2.4982e-3$	$2.5120e-3$	$1.7848e-3$	$1.4933e-3$	$1.3678e-3$	$2.1588e-3$
$D_X^{\times}$	$1.1101e+0$	$6.5647e+1$	$2.7827e+1$	$2.2760e+1$	$5.6485e+0$	$1.5933e+1$
$D_Y^{\times}$	$2.0676e+1$	$1.2477e+2$	$8.7552e+1$	$1.0619e+1$	$2.6020e+1$	$8.7162e+0$
$D_Z^{\times}$	$6.2437e+0$	$7.0604e+0$	$1.0871e+2$	$1.1127e+1$	–	–
$D_W^{\times}$	–	$7.2993e+1$	–	–	–	–
$D_P^{\times}$	–	–	–	–	$2.0251e+1$	$1.0069e+1$
$D_Q^{\times}$	–	–	–	–	$3.3421e+0$	$1.5636e+1$
k_1	$2.0262e+4$	$8.8493e+2$	$5.0718e+1$	$6.3584e-1$	$4.7041e-3$	$1.0483e+0$
k_1^r	$4.5700e+2$	–	–	–	–	–
k_2	$1.7084e+0$	$6.7929e+2$	$6.7135e+0$	$3.8833e+1$	–	–
k_2^r	$1.1334e-1$	–	–	–	–	–
k_3	–	$1.3432e+2$	–	–	–	–
k_4	–	$7.7769e-1$	–	–	–	–
c_1	–	–	–	–	$1.5411e+0$	$2.4232e+1$
B_T	140	415	107	136	94	152

Table 6. Internal Solute Concentrations at Stable States SS1 and SS2 in Figure 2 of paper. This information is graphically presented in Figure 3 of paper. All concentrations in M, and in scientific notation. Concentrations were found by dynamics simulation of vesicle reactor model until steady state was reached.

	**a(i)**	**a(ii)**	**b(i)**	**b(ii)**	**c(i)**	**c(ii)**
Stable Point 1						
x_1^*	$1.130e-3$	$4.502e-4$	$1.690e-3$	$8.010e-4$	$1.996e-3$	$6.552e-4$
y_1^*	$1.085e-3$	$1.623e-3$	$2.139e-3$	$3.033e-4$	$8.223e-4$	$2.008e-3$
z_1^*	$4.413e-3$	$3.122e-3$	$1.011e-3$	$1.511e-4$	–	–
w_1^*	–	$1.589e-3$	–	–	–	–
p_1^*	–	–	–	–	$1.723e-4$	$1.982e-3$
q_1^*	–	–	–	–	$1.896e-3$	$1.969e-3$

Table 6 . *Cont.*

	a(i)	a(ii)	b(i)	b(ii)	c(i)	c(ii)
Stable Point 2						
x_2^*	$7.664e-4$	$6.479e-5$	$1.055e-3$	$1.884e-4$	$1.790e-3$	$9.761e-5$
y_2^*	$5.156e-4$	$6.648e-4$	$4.495e-3$	$1.618e-3$	$8.669e-4$	$4.047e-3$
z_2^*	$6.363e-3$	$3.964e-3$	$1.439e-4$	$1.507e-3$	–	–
w_2^*	–	$3.491e-3$	–	–	–	–
p_2^*	–	–	–	–	$2.650e-5$	$1.146e-3$
q_2^*	–	–	–	–	$2.779e-3$	$2.238e-3$

7. Steady State: Non-Spherical Vesicle Morphology Examples

Only spherical vesicle shapes fulfilling steady state are depicted on Figure 2 of the paper, because the vesicle reactor model cannot admit any other morphology. However, if vesicle surface area were not directly determined by volume, but was a separate state variable, then there exist many non-spherical vesicle morphologies which could theoretically also sustain steady state (*i.e.*, deflated vesicles with surplus membrane surface area). Figure 2 shows prolate spheroid shapes for vesicles, which are possible shapes corresponding to points A–F in Figure 2 of the main paper.

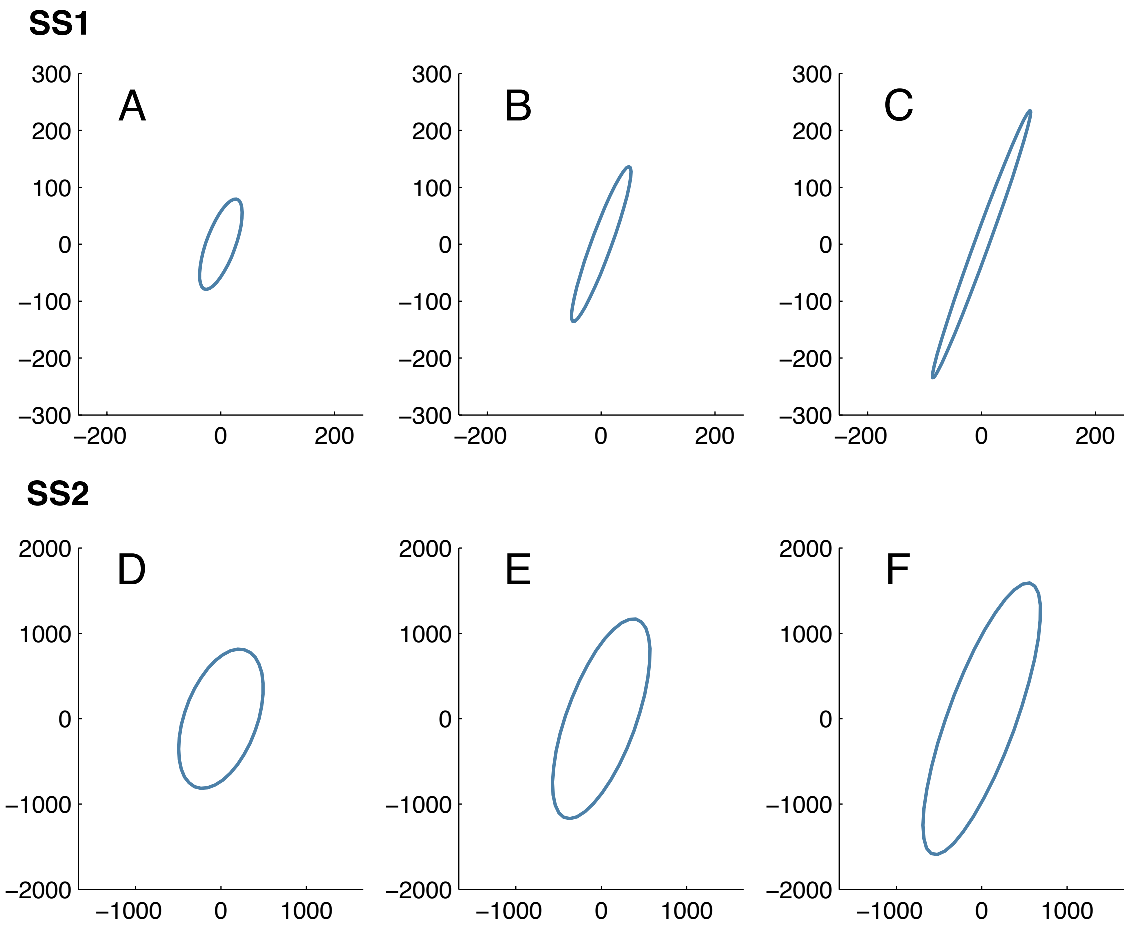

Figure 2. Examples of non-spherical vesicle morphologies. Axes in nm.

References and Notes

1. Computational note: To avoid multiplication of large numbers, it is better to perform e.g., $\Omega^{\oslash}(-k_1(X/\Omega^{\oslash})(Y/\Omega^{\oslash})(Y/\Omega^{\oslash}))$ rather than $-\frac{k_1}{(\Omega^{\oslash})^2}XY^2$.
2. Pawłowski, P.H.; Zielenkiewicz, P. Biochemical kinetics in changing volumes. *Acta Biochim. Pol.* **2004**, *51*, 231–243.
3. Mavelli, F.; Ruiz-Mirazo, K. Theoretical conditions for the stationary reproduction of model protocells. *Integr. Biol.* **2013**, *5*, 324–341.

© 2015 by the authors; licensee MDPI, Basel, Switzerland. This article is an open access article distributed under the terms and conditions of the Creative Commons Attribution license (http://creativecommons.org/licenses/by/4.0/).
